# Supplementary material for: Eliminating accidental deviations to minimize generalization error and maximize replicability: Applications in connectomics and genomics
Source: PLoS Comput Biol. 2021 Sep 16;17(9):e1009279. doi: 10.1371/journal.pcbi.1009279 (PMC8500408; doi:10.1371/journal.pcbi.1009279)
Supplement: S1 Text — (PDF) [file pcbi.1009279.s001.pdf]

Supporting Information 1: Eliminating accidental deviations to minimize generalization error and maximize replicability: applications in connectomics and genomics

Eric W. Bridgeford[1], Shangsi Wang[1], Zeyi Wang[1], Ting Xu[3], Cameron Craddock[3], Jayanta Dey[1], Gregory Kiar[1], William Gray-Roncal[1], Carlo Colantuoni[1], Christopher Douville[1], Stephanie Noble[4], Carey E. Priebe[1], Brian Caffo[1], Michael Milham[3], Xi-Nian Zuo[2,5], Consortium for Reliability and Reproducibility, Joshua T. Vogelstein[1,6*]

S1 Data Repeatability Statistics

Intraclass Correlation Coefficient The intraclass correlation coefficient (`ICC`) is a commonly used data replicability statistic [1]. The absolute agreement `ICC`, or `ICC(1,1)`, is the fraction of the total variability that is across-item variability, that is, `ICC` is defined as the across-item variability divided by the within-item plus across-item variability. `ICC` has several limitations. First, it is univariate, meaning if the data are multidimensional, they must first be represented by univariate statistics, thereby discarding multivariate information. This potentially makes `ICC` unsuitable when an informative univariate summary measure is unavailable or unknown, which is frequently the case in the high dimensional data that is the focus of this manuscript. Second, `ICC` is based on a Gaussian assumption characterizing the data. Thus, any deviations from this assumption may render the interpretation of the magnitude of `ICC` questionable, because non-Gaussian measurements that are highly replicable could potentially yield quite low `ICC` [2–4]. Third, the Intraclass correlation coefficient is highly sensitive to the design of the study [4, 5]; care must be taken to ensure that the form of ICC chosen accurately reflects the design of the study of interest. Further, `ICC` is substantially impacted by the presence of outliers in measurements [6]. Finally, there are numerous definitions of estimates of `ICC`[1] designed for different experimental setups, and researchers regularly use (and misuse) the different estimators in generic contexts [4, 7]. In practice, it is unclear the extent to which the use of inappropriate estimators of `ICC` is impactful [8].

Numerous multivariate generalizations of the `ICC` attempt to overcome the requirement of `ICC` to operate on univariate data. The Image Intra-Class Correlation (`I2C2`) was introduced to mitigate `ICC`'s univariate limitation [9]. Specifically, `I2C2` operates on covariances matrices, rather than variances. To obtain a univariate summary of replicability, `I2C2` operates on the trace of the covariance matrices, one of several possible strategies, similar to most multivariate analysis of variance procedures [10]. Thus, while overcoming one limitation of `ICC`, `I2C2` still heavily leverages Gaussian assumptions of the data to justify its validity. [11] highlight a number of limitations with using estimates of covariance in the context of assessing multivariate replicability. Chiefly, sampling variance of covariance components in the high dimensionality; low-sample-size (HDLSS) regime is problematic, which is an characteristic of increasing prevalence in biological data.

Fingerprinting Index The fingerprinting index [12, 13] provides a metric for quantifying individual connectivity profiles in resting-state MRI (fMRI). Specifically, the fingerprinting index operates on the pairwise correlation of the vectorized connectivity matrices. A high fingerprinting index corresponds to the connectivity matrices being most strongly correlated within-subject versus between-subject. An important clarification for fingerprinting is that the connectivity matrices must be more strongly correlated than *any other measurement* within a particular scanning session, otherwise the fingerprinting index

[1] Johns Hopkins University, Baltimore, Maryland, USA, [2] Shanghai Jiaotong University, Shanghai, China [3] Child Mind Institute, New York, New York, USA [4] Yale University, New Haven, Connecticut, USA [5] Beijing Normal University, Beijing, China, Nanning Normal University, Nanning, China, University of Chinese Academy of Sciences, Beijing, China, [6] Progressive Learning, Baltimore, Maryland, USA. * jovo@jhu.edu.

will be 0, as the fingerprinting index uses only the nearest-neighbor associated with a given item. Unlike the other strategies employed in this manuscript, the fingerprinting index produces a statistic for each possible ordering of 2 measurement sessions, that is, if each item is measured s times, fingerprinting produces $s(s-1)$ statistics. To enable fingerprinting for assessing the effectiveness of a strategy, we instead averaged across all $s(s-1)$ statistics, which will henceforth be referred to as `Fingerprinting`.

Kendall's Coefficient of Concordance Kendall's Coefficient of Concordance, or Kendall's W, is a univariate non-parametric statistic for assessing the extent to which multiple measurements of the same item agree. Like inter-item discriminability and the fingerprinting index, estimates of Kendall's W operate on the ranks of data. Specifically, Kendall's W computes the total rank of all measurements associated with a single item, and compares an item's total rank to the average value of the total rank. An important consideration is that Kendall's W operates directly on the measurements themselves, rather than on scalar summary measures of the relationships amongst the measurements. As such, Kendall's W cannot be applied directly to data that is inherently multivariate using traditional methods of ranking. For this reason, we do not formally evaluate Kendall's W within the context of this manuscript.

Kernel Methods Maximum mean discrepancy (`MMD`) [14] provides a non-parametric framework for comparing whether two samples are drawn from the same distribution. `MMD` subverts Gaussian assumptions by embedding the points in a reproducing kernel Hilbert Space (RKHS), and looking for functions over the unit ball in the RKHS which maximize the difference in the means of the embedded points. In the two-item regime, `MMD` can be shown to be equivalent to the Hilbert-Schmidt Independence Criterion (`HSIC`) [15–17], which provides a natural generalization of `MMD` when the number of classes exceeds two. To date, to our knowledge, there does not exist a k-sample variant of `MMD`.

Distance Components (`DISCO`) [18] extends the classical Analysis of Variance (`ANOVA`) framework to cases where the distributions are not necessarily Gaussian. In contrast to `ANOVA` which makes simplifying assumptions of normality, `DISCO` operates on the dispersion of the samples based on the Euclidean Distance, comparing the within-class dispersion to the between-class dispersion. `DISCO` produces a consistent test against general alternatives as the number of observations s per item goes to infinity. [19] shows a closed form relationship between `Kernel` and other Energy statistics approaches, such as Distance correlation. The result is that using Distance correlation for k-sample testing results in a test statistic that has bias relative to the `Kernel` statistic, but will yield the same p-value. Further, [19] shows the equivalence between Distance correlation and `HSIC/MMD`. Thus, in this manuscript, we use `Kernel` to refer to either `DISCO` or `MMD` as appropriate. In all cases, we use the default kernel, which is the Gaussian kernel with the typical bandwidth specification, as implemented in the `kernlab` package [20] (`MMD`) and `energy` (`DISCO`) package [21]. Note that in many real data scenarios, s is small (particularly, most "repeat measurements" datasets have $s = 2$), and the finite-sample performance of `Kernel` on such a small number of repeat trials is not known.

References

1. Shrout PE, Fleiss JL. Intraclass correlations: uses in assessing rater reliability. Psychol Bull. 1979 Mar;86(2):420–428.
2. Mehta S, Bastero-Caballero RF, Sun Y, Zhu R, Murphy DK, Hardas B, et al. Performance of intraclass correlation coefficient (ICC) as a reliability index under various distributions in scale reliability studies. Stat Med. 2018 Aug;37(18):2734–2752.
3. Ten Cate DF, Luime JJ, Hazes JMW, Jacobs JWG, Landewé R. Does the intraclass correlation coefficient always reliably express reliability? Comment on the article by Cheung et al. Arthritis Care Res. 2010 Sep;62(9):1357–8; author reply 1358.
4. Bobak CA, Barr PJ, O'Malley AJ. Estimation of an inter-rater intra-class correlation coefficient that overcomes common assumption violations in the assessment of health measurement scales. BMC Med Res Methodol. 2018 Sep;18(1):93.

5. Koo TK, Li MY. A Guideline of Selecting and Reporting Intraclass Correlation Coefficients for Reliability Research. J Chiropr Med. 2016 Jun;15(2):155–163.
6. Vaz S, Falkmer T, Passmore AE, Parsons R, Andreou P. The Case for Using the Repeatability Coefficient When Calculating Test–Retest Reliability. PLoS One. 2013;8(9).
7. Bartko JJ. On various intraclass correlation reliability coefficients. Psychol Bull. 1976;.
8. Chen G, Taylor PA, Haller SP, Kircanski K, Stoddard J, Pine DS, et al. Intraclass correlation: Improved modeling approaches and applications for neuroimaging. Hum Brain Mapp. 2018 Mar;39(3):1187–1206.
9. Shou H, Eloyan A, Lee S, Zipunnikov V, Crainiceanu A, Nebel M, et al. Quantifying the reliability of image replication studies: the image intraclass correlation coefficient (I2C2). Cognitive, Affective, & Behavioral Neuroscience. 2013;13(4):714–724.
10. Huberty CJ, Olejnik S. Applied MANOVA and Discriminant Analysis. John Wiley & Sons; 2006.
11. Webb NM, Shavelson RJ, Haertel EH. 4 Reliability Coefficients and Generalizability Theory. In: Rao CR, Sinharay S, editors. Handbook of Statistics. vol. 26. Elsevier; 2006. p. 81–124.
12. Finn ES, Shen X, Scheinost D, Rosenberg MD, Huang J, Chun MM, et al. Functional connectome fingerprinting: identifying individuals using patterns of brain connectivity. Nat Neurosci. 2015 Nov;18(11):1664–1671.
13. Finn ES, Scheinost D, Finn DM, Shen X, Papademetris X, Constable RT. Can brain state be manipulated to emphasize individual differences in functional connectivity? Neuroimage. 2017 Oct;160:140–151.
14. Gretton A, Borgwardt KM, Rasch MJ, Schölkopf B, Smola A. A Kernel Two-Sample Test. Journal of Machine Learning Research. 2012;13(Mar):723–773. Available from: http://jmlr.csail.mit.edu/papers/v13/gretton12a.html.
15. Sejdinovic D, Sriperumbudur B, Gretton A, Fukumizu K. Equivalence of distance-based and RKHS-based statistics in hypothesis testing. arXiv. 2012 Jul;.
16. Shen C, Priebe CE, Vogelstein JT. The Exact Equivalence of Independence Testing and Two-Sample Testing. arXiv. 2019 Oct;Available from: https://arxiv.org/abs/1910.08883.
17. Shen C, Vogelstein JT. The Exact Equivalence of Distance and Kernel Methods for Hypothesis Testing. arXiv. 2018 Jun;Available from: https://arxiv.org/abs/1806.05514.
18. Rizzo ML, Székely GJ, et al. Disco analysis: A nonparametric extension of analysis of variance. The Annals of Applied Statistics. 2010;4(2):1034–1055.
19. Shen C, Vogelstein JT. The exact equivalence of distance and kernel methods for hypothesis testing. arXiv preprint arXiv:180605514. 2018;.
20. Karatzoglou A, Smola A, Hornik K, Zeileis A. kernlab – An S4 Package for Kernel Methods in R. Journal of Statistical Software. 2004;11(9):1–20. Available from: http://www.jstatsoft.org/v11/i09/.
21. Rizzo M, Sz/'ekely G. E-Statistics: Multivariate Inference via the Energy of Data [R package energy version 1.7-7]. Comprehensive R Archive Network (CRAN);.
