# Supplementary material for: Eliminating accidental deviations to minimize generalization error and maximize replicability: Applications in connectomics and genomics
Source: PLoS Comput Biol. 2021 Sep 16;17(9):e1009279. doi: 10.1371/journal.pcbi.1009279 (PMC8500408; doi:10.1371/journal.pcbi.1009279)
Supplement: S2 Text — (PDF) [file pcbi.1009279.s002.pdf]

Supporting Information 2: Eliminating accidental deviations to minimize generalization error and maximize replicability: applications in connectomics and genomics

Eric W. Bridgeford[1], Shangsi Wang[1], Zeyi Wang[1], Ting Xu[3], Cameron Craddock[3], Jayanta Dey[1], Gregory Kiar[1], William Gray-Roncal[1], Carlo Colantuoni[1], Christopher Douville[1], Stephanie Noble[4], Carey E. Priebe[1], Brian Caffo[1], Michael Milham[3], Xi-Nian Zuo[2,5], Consortium for Reliability and Reproducibility, Joshua T. Vogelstein[1,6*]

S2 Population and Sample **`Discr`** Suppose that $\boldsymbol{\theta}_i \in \boldsymbol{\Theta}$ represents a physical property of interest for a particular item i. In a biological context, for instance, an item could be a participant in a study, and the property of interest could be the individual's true brain network, or connectome. We cannot directly observe the physical property, but rather, we must first measure $\boldsymbol{\theta}_i$ and then "wrangle" it. Call the measurement function, $f \in \mathcal{F}$ for a family of possible measurement functions $\mathcal{F}$ That is, $f : \boldsymbol{\Theta} \rightarrow \boldsymbol{\mathcal{W}}$. So, measurements of $\boldsymbol{\theta}_i$ are observed as $f(\boldsymbol{\theta}_i) = \boldsymbol{w}_i$. However, $\boldsymbol{w}_i$ may be a noisy, with measurement artefacts. Alternately, $\boldsymbol{w}_i$ might not be the property of interest, for example, if the property is a network, perhaps $\boldsymbol{w}_i$ is a multivariate time-series, from which we can estimate a network. We therefore have another function, $g \in \mathcal{G} : \boldsymbol{\mathcal{W}} \rightarrow \boldsymbol{\mathcal{X}}$, which represents the data wrangling procedure to take the measurement and produce an informative derivative (for instance, confound removal). The family of possible data wrangling procedures to produce the informative derivative is $\mathcal{G}$. In this fashion, the output of interest is $\boldsymbol{x}_i = g(f(\boldsymbol{\theta}_i))$.

The goal of experimental design is to choose an f and g that yield high-quality and useful inferences, that is, that yield $\boldsymbol{x}$'s that we can use for various inferential purposes. When we have repeated measurements of the same items, we can use those samples to our advantage. Given $\boldsymbol{x}_i^j$, which is the jth measurement of sample i, we would expect $\boldsymbol{x}_i^j$ to be more similar to $\boldsymbol{x}_i^{j'}$ (another measurement of the same item), than to any measurement of a different item $\boldsymbol{x}_{i'}^{j''}$. Formally, let $\delta : \boldsymbol{\mathcal{X}} \times \boldsymbol{\mathcal{X}} \rightarrow [0, \infty)$ be a distance metric, we define the population `Discr`:

$$D_{\delta,f,g} = \mathbb{P}\Big(\delta(\boldsymbol{x}_i^j, \boldsymbol{x}_i^{j'}) < \delta(\boldsymbol{x}_i^j, \boldsymbol{x}_{i'}^{j''})\Big)$$

That is, "population `Discr`" D represents the average probability that the *within-item distance* $\delta(\boldsymbol{x}_i^j, \boldsymbol{x}_i^{j'})$ is less than the *between-item distance* $\delta(\boldsymbol{x}_i^j, \boldsymbol{x}_{i'}^{j''})$. `Discr` depends on the choice of distance δ, as well as the measurement protocal f and the analysis choices g.

The population `Discr` represents a property of the distribution of $\boldsymbol{\theta}_i$. In real data since we do not observe the true distribution, we instead rely on the sample `Discr`. Suppose a dataset consists of $i \in \{1, \dots, n\}$ items, where each item i has J_i repeat measurements. The sample `Discr` is defined:

$$\texttt{Discr}\left\{\boldsymbol{x}_i^j\right\}_{j\in[J_i], i\in[n]} = \frac{\sum_{i\in[n]}\sum_{j\in[J_i]}\sum_{j'\neq j}\sum_{i'\neq i}\sum_{j''\in[J_{i'}]}\left(\mathbb{1}_{\left\{\delta(\boldsymbol{x}_i^j, \boldsymbol{x}_i^{j'}) < \delta(\boldsymbol{x}_i^j, \boldsymbol{x}_{i'}^{j''})\right\}}\right)}{\sum_{i\in[n]}\sum_{j\in[J_i]}\sum_{j'\neq j}\sum_{i'\neq i}\sum_{j''\in[J_{i'}]} 1}.$$

It can be shown [1] that the under the multivariate additive noise model in Assumption 1, that the sample `Discr` is both a consistent and unbiased estimator for population `Discr`.

[1] Johns Hopkins University, Baltimore, Maryland, USA, [2] Shanghai Jiaotong University, Shanghai, China [3] Child Mind Institute, New York, New York, USA [4] Yale University, New Haven, Connecticut, USA [5] Beijing Normal University, Beijing, China, Nanning Normal University, Nanning, China, University of Chinese Academy of Sciences, Beijing, China, [6] Progressive Learning, Baltimore, Maryland, USA. * jovo@jhu.edu.

References

1. Wang Z, Bridgeford E, Wang S, Vogelstein JT, Caffo B. Statistical Analysis of Data Repeatability Measures. arXiv. 2020 May;Available from: https://arxiv.org/abs/2005.11911v3.
