# Supplementary material for: Eliminating accidental deviations to minimize generalization error and maximize replicability: Applications in connectomics and genomics
Source: PLoS Comput Biol. 2021 Sep 16;17(9):e1009279. doi: 10.1371/journal.pcbi.1009279 (PMC8500408; doi:10.1371/journal.pcbi.1009279)
Supplement: S3 Text — (PDF) [file pcbi.1009279.s003.pdf]

Supporting Information 3: Eliminating accidental deviations to minimize generalization error and maximize replicability: applications in connectomics and genomics

Eric W. Bridgeford[1], Shangsi Wang[1], Zeyi Wang[1], Ting Xu[3], Cameron Craddock[3], Jayanta Dey[1], Gregory Kiar[1], William Gray-Roncal[1], Carlo Colantuoni[1], Christopher Douville[1], Stephanie Noble[4], Carey E. Priebe[1], Brian Caffo[1], Michael Milham[3], Xi-Nian Zuo[2,5], Consortium for Reliability and Reproducibility, Joshua T. Vogelstein[1,6*]

S3 `Discr` Provides an Informative Bound for Inference During experimental design, the extent of subsequent inference tasks may be unknown. A natural question may be, what are the implications of the selection of a discriminable experimental design? Formally, assume the task of interest is binary classification: that is, $\mathcal{Y} = \{0, 1\}$, and we seek a classifier $h : \mathcal{X} \to \mathcal{Y}$. The goal of experimental design in this context is to choose the options (f^*, g^*) that will minimize the classification loss:

$$(f^*, g^*) = \underset{(f,g)\in\mathcal{F}\times\mathcal{G}}{\operatorname{argmin}} \mathbb{P}(h(\boldsymbol{x}) \neq y | \boldsymbol{x} = f(g(\boldsymbol{\theta}))).$$

For a fixed (f, g), the minimal prediction error is achieved by the Bayes optimal classifier [1]:

$$h_x^*(\boldsymbol{x}) \triangleq \underset{y\in\{0,1\}}{\operatorname{argmax}} \mathbb{P}\big(y_i = y \big| \boldsymbol{x}\big)\pi_y \tag{1}$$

$$= \underset{y\in\{0,1\}}{\operatorname{argmax}} \log \mathbb{P}\big(y_i = y\big|\boldsymbol{x}\big) + \log \pi_y, \tag{2}$$

where $\pi_y = \mathbb{P}(y_i = y)$, and let L_x^* denote the error of the Bayes optimal classifier; that is, the error achieved by h_x^*.

Assumption 1 (Multivariate Additive Noise Setting).

The multivariate additive noise setting can be described as follows. For items $i = 1, \ldots, n$ and sessions $j = 1, \ldots, s$:

$$\begin{aligned} y_i &\overset{iid}{\sim} Bern(\pi_1), \\ \boldsymbol{\theta}_i &\overset{ind}{\sim} \mathcal{F}(\boldsymbol{\mu}_{y_i}, \boldsymbol{\Sigma}_\theta), \\ \boldsymbol{\epsilon}_i^j &\overset{iid}{\sim} \mathcal{F}(\boldsymbol{c}, \boldsymbol{\Sigma}_\epsilon) \text{ independent of } \boldsymbol{\theta}_i, \\ \boldsymbol{x}_i^j &= \boldsymbol{\theta}_i + \boldsymbol{\epsilon}_i^j = f(g(\boldsymbol{\theta}_i)). \end{aligned}$$

where $\mathcal{F}(\boldsymbol{\mu}, \boldsymbol{\Sigma})$ denotes a distribution with a finite mean vector $\boldsymbol{\mu}$ and a finite, non-singular covariance $\boldsymbol{\Sigma}$.

To connect the above model more directly with Eq. (1), we can let look at a special case

$$f(\boldsymbol{\theta}_i) = \boldsymbol{\theta}_i + \boldsymbol{\eta}_i^j, \qquad g(f(\boldsymbol{\theta}_i)) = \boldsymbol{\theta}_i + \boldsymbol{\eta}_i^j + \boldsymbol{\tau}_i^j, \qquad \boldsymbol{\epsilon}_i^j = \boldsymbol{\eta}_i^j + \boldsymbol{\tau}_i^j,$$

where we assume that $\boldsymbol{\eta}_i^j \perp\!\!\!\perp \boldsymbol{\tau}_i^j$, and both $\boldsymbol{\eta}_i^j$ and $\boldsymbol{\tau}_i^j$ are multivariate Gaussian. Using Bayes rule and Assumption 1, note that the probability that an observation $\boldsymbol{x}_i^j$ is from class y is given by:

$$\mathbb{P}\big(y_i = y\big|\boldsymbol{x}\big) = \frac{\mathbb{P}\big(\boldsymbol{x}\big|y_i = y\big)\mathbb{P}(y_i = y)}{\mathbb{P}(\boldsymbol{x})}$$

[1] Johns Hopkins University, Baltimore, Maryland, USA, [2] Shanghai Jiaotong University, Shanghai, China [3] Child Mind Institute, New York, New York, USA [4] Yale University, New Haven, Connecticut, USA [5] Beijing Normal University, Beijing, China, Nanning Normal University, Nanning, China, University of Chinese Academy of Sciences, Beijing, China, [6] Progressive Learning, Baltimore, Maryland, USA. * jovo@jhu.edu.

$$\Rightarrow \log \mathbb{P}\big(y_i = y \big| \boldsymbol{x}\big) \propto -\frac{1}{2}(\boldsymbol{x}-\boldsymbol{\mu}_y)^\top \boldsymbol{\Sigma}_x(\boldsymbol{x}-\boldsymbol{\mu}_y) + \log(\pi_y)$$

where $\boldsymbol{\Sigma}_x = \boldsymbol{\Sigma}_\theta + \boldsymbol{\Sigma}_\epsilon$ is constant between the two classes (that is, the variance is homoscedastic), and y is a generic value in $\{0,1\}$ that a realization y_i can take. This follows directly by taking the log of the density function of the multivariate normal distribution, and removing terms not proportional in y. The Bayes optimal classifier is:

$$h_x^*(\boldsymbol{x}) = \underset{y\in\{0,1\}}{\operatorname{argmax}}\Big[-\frac{1}{2}(\boldsymbol{x}-\boldsymbol{\mu}_y)\boldsymbol{\Sigma}_x(\boldsymbol{x}-\boldsymbol{\mu}_y) + \log \pi_y\Big].$$

In the general case, the Bayes optimal error can be computed explicitly using that:

$$L_x^* \triangleq \mathbb{E}\big[\mathbb{1}_{h_x^*(\boldsymbol{x})\neq y}\big] = \sum_{y\in\{0,1\}} \int_{\mathcal{X}} \mathbb{P}(h_x^*(\boldsymbol{x}) \neq y|\boldsymbol{x})\mathbb{P}(\boldsymbol{x})\, \mathrm{d}\boldsymbol{x},$$

using standard rules of integration. Even when the true class distributions are known, however, computation of this integral explicitly tends to be rather tedious. For this reason, much work is dedicated to identifying cases in which the Bayes error can be bounded.

Importantly, the Bayes error can, in fact, be upper bounded by a decreasing function of `Discr`, as shown in the theorem below. In words, this theorem specifies the desirability of high `Discr`: a higher discriminability results in a lower bound on the error of future inferential tasks. Correspondingly, a strategy with a higher discriminability will have a lower bound on the error than another strategy with a lower discriminability.

Theorem 2. *Let $\Big\{(\boldsymbol{x}_i^j, y_i) : j \in [s]\Big\}_{i\in[n]}$ follow the multivariate additive noise setting, given in Assumption 1. Then there exists a decreasing function $\gamma(\cdot)$ of the discriminability D where:*

$$L_{f,g}^* \leq \gamma(D_{f,g})$$

where L^* is the Bayes error, or the error achieved by the Bayes optimal classifier $h_{f,g}^*(\boldsymbol{\theta}_i)$.

Proof of Theorem (2).
Consider the additive noise setting, that is $\boldsymbol{x}_i^j = \boldsymbol{\theta}_i + \boldsymbol{\epsilon}_i^j$,

$$\begin{aligned}
D &= \mathbb{P}\big(\delta_{i,j,j'} < \delta_{i,i',j,j''}\big)\\
&= \mathbb{P}(\|\boldsymbol{x}_i^j - \boldsymbol{x}_i^{j'}\| < \|\boldsymbol{x}_i^j - \boldsymbol{x}_{i'}^{j''}\|)\\
&= \mathbb{P}(\|\boldsymbol{\epsilon}_i^j - \boldsymbol{\epsilon}_i^{j'}\| < \|\boldsymbol{\theta}_i + \boldsymbol{\epsilon}_i^j - \boldsymbol{\theta}_{i'} - \boldsymbol{\epsilon}_{i'}^{j''}\|)\\
&\leq \mathbb{P}(\|\boldsymbol{\epsilon}_i^j - \boldsymbol{\epsilon}_i^{j'}\| < \|\boldsymbol{\theta}_i - \boldsymbol{\theta}_{i'}\| + \|\boldsymbol{\epsilon}_i^j - \boldsymbol{\epsilon}_{i'}^{j''}\|)\\
&= \mathbb{P}(\|\boldsymbol{\epsilon}_i^j - \boldsymbol{\epsilon}_i^{j'}\| - \|\boldsymbol{\epsilon}_i^j - \boldsymbol{\epsilon}_{i'}^{j''}\| < \|\boldsymbol{\theta}_i - \boldsymbol{\theta}_{i'}\|)\\
&= \frac{1}{2}\mathbb{P}(\|\boldsymbol{\epsilon}_i^j - \boldsymbol{\epsilon}_i^{j'}\| - \|\boldsymbol{\epsilon}_i^j - \boldsymbol{\epsilon}_{i'}^{j''}\| < \|\boldsymbol{\theta}_i - \boldsymbol{\theta}_{i'}\| | \|\boldsymbol{\epsilon}_i^j - \boldsymbol{\epsilon}_i^{j'}\| - \|\boldsymbol{\epsilon}_i^j - \boldsymbol{\epsilon}_{i'}^{j''}\| < 0) +\\
&\quad \frac{1}{2}\mathbb{P}(\|\boldsymbol{\epsilon}_i^j - \boldsymbol{\epsilon}_i^{j'}\| - \|\boldsymbol{\epsilon}_i^j - \boldsymbol{\epsilon}_{i'}^{j''}\| < \|\boldsymbol{\theta}_i - \boldsymbol{\theta}_{i'}\| | \|\boldsymbol{\epsilon}_i^j - \boldsymbol{\epsilon}_i^{j'}\| - \|\boldsymbol{\epsilon}_i^j - \boldsymbol{\epsilon}_{i'}^{j''}\| > 0)\\
&= \frac{1}{2} + \frac{1}{2}\mathbb{P}(\|\boldsymbol{\epsilon}_i^j - \boldsymbol{\epsilon}_i^{j'}\| - \|\boldsymbol{\epsilon}_i^j - \boldsymbol{\epsilon}_{i'}^{j''}\| < \|\boldsymbol{\theta}_i - \boldsymbol{\theta}_{i'}\| | \|\boldsymbol{\epsilon}_i^j - \boldsymbol{\epsilon}_i^{j'}\| - \|\boldsymbol{\epsilon}_i^j - \boldsymbol{\epsilon}_{i'}^{j''}\| > 0)\\
&= \frac{1}{2} + \frac{1}{2}\mathbb{P}(\big|\|\boldsymbol{\epsilon}_i^j - \boldsymbol{\epsilon}_i^{j'}\| - \|\boldsymbol{\epsilon}_i^j - \boldsymbol{\epsilon}_{i'}^{j''}\|\big| < \|\boldsymbol{\theta}_i - \boldsymbol{\theta}_{i'}\|)\\
&= 1 - \frac{1}{2}\mathbb{P}(\big|\|\boldsymbol{\epsilon}_i^j - \boldsymbol{\epsilon}_i^{j'}\| - \|\boldsymbol{\epsilon}_i^j - \boldsymbol{\epsilon}_{i'}^{j''}\|\big| > \|\boldsymbol{\theta}_i - \boldsymbol{\theta}_{i'}\|).
\end{aligned}$$

To bound the probability above, we bound the $\|\boldsymbol{\theta}_i - \boldsymbol{\theta}_{i'}\|$ and $\big|\|\boldsymbol{\epsilon}_i^j - \boldsymbol{\epsilon}_i^{j'}\| - \|\boldsymbol{\epsilon}_i^j - \boldsymbol{\epsilon}_{i'}^{j''}\|\big|$ separately. We start with the first term

$$\mathbb{E}(\|\boldsymbol{\theta}_i - \boldsymbol{\theta}_{i'}\|^2) = \mathbb{E}(\boldsymbol{\theta}_i^T\boldsymbol{\theta}_i + \boldsymbol{\theta}_{i'}^T\boldsymbol{\theta}_{i'} - 2\boldsymbol{\theta}_i^T\boldsymbol{\theta}_{i'}) = 2\sigma_2^2.$$

Here, $\sigma_2^2 = \mathrm{tr}(\boldsymbol{\Sigma}_\theta)$ is the trace of covariance matrix of $\boldsymbol{\theta}_i$. We can apply Markov's Inequality for any $t > 0$:

$$\mathbb{P}(\|\boldsymbol{\theta}_i - \boldsymbol{\theta}_{i'}\| < t) \geq 1 - \frac{2\sigma_2^2}{t^2}. \tag{3}$$

Let a and b be two constants satisfying:

$$\mathbb{E}(\big|\|\boldsymbol{\epsilon}_i^j - \boldsymbol{\epsilon}_i^{j'}\| - \|\boldsymbol{\epsilon}_i^j - \boldsymbol{\epsilon}_{i'}^{j''}\|\big|^2) \geq a^2\sigma_\epsilon^2,$$
$$\frac{\mathbb{E}^2(\big|\|\boldsymbol{\epsilon}_i^j - \boldsymbol{\epsilon}_i^{j'}\| - \|\boldsymbol{\epsilon}_i^j - \boldsymbol{\epsilon}_{i'}^{j''}\|\big|^2)}{\mathbb{E}(\big|\|\boldsymbol{\epsilon}_i^j - \boldsymbol{\epsilon}_i^{j'}\| - \|\boldsymbol{\epsilon}_i^j - \boldsymbol{\epsilon}_{i'}^{j''}\|\big|)^4} \geq b$$

Furthermore, let $t^2 = \sqrt{2}a\sigma_\epsilon\sigma_\theta$, and define:

$$\theta = \frac{t^2}{\mathbb{E}(\big|\|\boldsymbol{\epsilon}_i^j - \boldsymbol{\epsilon}_i^{j'}\| - \|\boldsymbol{\epsilon}_i^j - \boldsymbol{\epsilon}_{i'}^{j''}\|\big|^2)} \leq \frac{\sqrt{2}a\sigma_\epsilon\sigma_\theta}{a^2\sigma_\epsilon^2} = \frac{\sqrt{2}\sigma_\theta}{a\sigma_\epsilon}.$$

If $a^2\sigma_\epsilon^2 \geq 2\sigma_\theta^2$, then $\theta \leq 1$. According to the Paley-Zygmund Inequality [2], that is:

$$\mathbb{P}(Z > \theta\mathbb{E}[Z]) \geq (1-\theta)^2\frac{\mathbb{E}[Z]^2}{\mathbb{E}[Z^2]}$$

for all $0 \leq \theta \leq 1$ and $Z \geq 0$, we can plug in the θ above to achieve

$$\mathbb{P}(\big|\|\boldsymbol{\epsilon}_i^j - \boldsymbol{\epsilon}_i^{j'}\| - \|\boldsymbol{\epsilon}_i^j - \boldsymbol{\epsilon}_{i'}^{j''}\|\big|^2 > t^2) \geq b\left(1 - \frac{t^2}{a^2\sigma_\epsilon^2}\right)^2 = b\left(1 - \frac{\sqrt{2}\sigma_\theta}{a\sigma_\epsilon}\right)^2.$$

Plugging t^2 into the inequality in Equation (3), we have:

$$\mathbb{P}(\|\boldsymbol{\theta}_i - \boldsymbol{\theta}_{i'}\|^2 < t^2) \geq 1 - \frac{2\sigma_\theta^2}{t^2} = 1 - \frac{\sqrt{2}\sigma_\theta}{a\sigma_\epsilon}.$$

Given that $\boldsymbol{\theta}_i$'s and $\boldsymbol{\epsilon}_i^j$'s are independent by supposition, we can combine the two inequalities:

$$\begin{aligned}
D &= \mathbb{P}(\delta_{i,t,t'} < \delta_{i,i',t,t''}) \\
&= \mathbb{P}(\|\boldsymbol{x}_i^j - \boldsymbol{x}_i^{j'}\| < \|\boldsymbol{x}_i^j - \boldsymbol{x}_{i'}^{j''}\|) \\
&\leq 1 - \frac{1}{2}\mathbb{P}(\big|\|\boldsymbol{\epsilon}_i^j - \boldsymbol{\epsilon}_i^{j'}\| - \|\boldsymbol{\epsilon}_i^j - \boldsymbol{\epsilon}_{i'}^{j''}\|\big| > \|\boldsymbol{\theta}_i - \boldsymbol{\theta}_{i'}\|) \\
&\leq 1 - \frac{1}{2}\mathbb{P}(\big|\|\boldsymbol{\epsilon}_i^j - \boldsymbol{\epsilon}_i^{j'}\| - \|\boldsymbol{\epsilon}_i^j - \boldsymbol{\epsilon}_{i'}^{j''}\|\big|^2 > t^2)P(\|\boldsymbol{\theta}_i - \boldsymbol{\theta}_{i'}\|^2 < t^2) \\
&\leq 1 - \frac{1}{2}b\left(1 - \frac{\sqrt{2}\sigma_\theta}{a\sigma_\epsilon}\right)^3
\end{aligned}$$

Note that the resulted bound holds true even if $a^2\sigma_\epsilon^2 < 2\sigma_\theta^2$, as the right hand side becomes greater than 1. This produces a bound for $\frac{\sigma_\theta}{\sigma_\epsilon}$:

$$\frac{\sigma_\theta}{\sigma_\epsilon} \geq \frac{a}{\sqrt{2}}\left(1 - \left(\frac{2-2D}{b}\right)^{1/3}\right). \tag{4}$$

To obtain a bound on Bayes error, we use the following two observations:

1. The weighted covariance matrix of the measurements is non-singular: Define $\mathbf{\Sigma}_x$ as the weighted covariance matrix of $\boldsymbol{x}$:

$$\begin{aligned}\mathbf{\Sigma}_x &= \pi_0 \text{Var}(\boldsymbol{x}_i^j|\boldsymbol{y}_i = 0) + \pi_1 \text{Var}(\boldsymbol{x}_i^j|\boldsymbol{y}_i = 1) \\ &= \pi_0 \text{Var}(\boldsymbol{\theta}_i|\boldsymbol{y}_i = 0) + \pi_1 \text{Var}(\boldsymbol{\theta}_i|\boldsymbol{y}_i = 1) + \text{Var}(\boldsymbol{\epsilon}_i^j) \\ &= \mathbf{\Sigma}_\theta + \mathbf{\Sigma}_\epsilon.\end{aligned}$$

   which follows since $\pi_0 + \pi_1 = 1$. Further, note that since both $\mathbf{\Sigma}_\theta$ and $\mathbf{\Sigma}_\epsilon$ are finite and non-singular, their sum $\mathbf{\Sigma}_x$ is also finite and non-singular.
2. The between-class difference is finite: Denote $\Delta\boldsymbol{\mu}$ to be the difference between the means of the two classes. Since $\boldsymbol{\epsilon}_i^j$ is assumed to be independent of $\boldsymbol{y}_i$:

$$\Delta\boldsymbol{\mu} = \mathbb{E}(\boldsymbol{x}_i^j|\boldsymbol{y}_i = 0) - \mathbb{E}(\boldsymbol{x}_i^j|\boldsymbol{y}_i = 1) = \mathbb{E}(\boldsymbol{\theta}_i|\boldsymbol{y}_i = 0) - \mathbb{E}(\boldsymbol{\theta}_i|\boldsymbol{y}_i = 1).$$

We apply Devijver and Kittler's result [3], from equation (2.93), which gives that:

$$L^* \leq \frac{2\pi_0\pi_1}{1 + \pi_0\pi_1 \Delta\boldsymbol{\mu}^\top \mathbf{\Sigma}_x^{-1} \Delta\boldsymbol{\mu}}.$$

Denote $\mathbf{\Sigma}' = \frac{1}{\sigma_\epsilon^2}\mathbf{\Sigma}_\epsilon$. By inequality (4), note that $\sigma_\epsilon^2 \leq \sigma_{\epsilon*}^2(D)$, where:

$$\sigma_{\epsilon*}(D) = \frac{\sqrt{2}\sigma_\theta}{a(1 - (\frac{2-2D}{b})^{1/3})}.$$

Hence, $\mathbf{\Sigma}_x \preceq \mathbf{\Sigma}_*(D)$ where:

$$\mathbf{\Sigma}_*(D) = \mathbf{\Sigma}_\theta + \sigma_{\epsilon*}^2 \mathbf{\Sigma}'.$$

Therefore, $\mathbf{\Sigma}_x^{-1} \succeq \mathbf{\Sigma}_*^{-1}(D)$, and we obtain:

$$L^* \leq \frac{2\pi_0\pi_1}{1 + \pi_0\pi_1 \Delta\boldsymbol{\mu}^\top \mathbf{\Sigma}_x^{-1} \Delta\boldsymbol{\mu}} \leq \frac{2\pi_0\pi_1}{1 + \pi_0\pi_1 \Delta\boldsymbol{\mu}^\top \mathbf{\Sigma}_*^{-1}(D) \Delta\boldsymbol{\mu}} = \gamma(D). \quad \blacksquare$$

where $\gamma(D) = \frac{2\pi_0\pi_1}{1+\pi_0\pi_1 \Delta\boldsymbol{\mu}^\top \mathbf{\Sigma}_*^{-1}(D)\Delta\boldsymbol{\mu}}$ is decreasing in D.

Next, we will generalize this theorem to a broader class of stochastic measurements. A local ordinal embedding [4] $\varphi : \mathcal{X} \to \mathcal{W}$ with respect to a pair of distance metrics δ_x, δ_w for a set of measurements $X = \{\boldsymbol{x}_i\}_{i \in [n]}$ is defined as a function where if $\boldsymbol{x}_i, \boldsymbol{x}_{i'}, \boldsymbol{x}_j, \boldsymbol{x}_{j'} \in X$, then:

$$\delta_x(\boldsymbol{x}_i, \boldsymbol{x}_{i'}) < \delta_x(\boldsymbol{x}_j, \boldsymbol{x}_{j'}) \Rightarrow \delta_w(\varphi(\boldsymbol{x}_i), \varphi(\boldsymbol{x}_{i'})) < \delta_w(\varphi(\boldsymbol{x}_j), \varphi(\boldsymbol{x}_{j'}))$$

Effectively, the statement asserts that if a pair of points are closer than another pair of points, than the pair of embedded points are closer than the other pair of embedded points. In other words, the *ordering of distances* is preserved after embedding with φ. While this fairly broad class of embeddings preserves discriminability rather trivially, in fact, an even broader class embeddings will further preserve discriminability. In particular, an embedding need only preserve *within-item* distance orderings, rather than *all pairs* of distances. We define this class of embeddings as a **within-item** ordinal embedding. Suppose that $X = \left\{\boldsymbol{x}_i^j : j \in [s]\right\}_{i \in [n]}$ denotes a set of measurements of n individuals, measured s times each. If $\boldsymbol{x}_i^j, \boldsymbol{x}_i^{j'}, \boldsymbol{x}_{i'}^{j''} \in X$, then:

$$\delta_x(\boldsymbol{x}_i^j, \boldsymbol{x}_i^{j'}) < \delta_x(\boldsymbol{x}_i^j, \boldsymbol{x}_{i'}^{j''}) \Rightarrow \delta_w\Big(\varphi(\boldsymbol{x}_i^j), \varphi(\boldsymbol{x}_i^{j'})\Big) < \delta_w\Big(\varphi(\boldsymbol{x}_i^j), \varphi(\boldsymbol{x}_{i'}^{j''})\Big)$$

This class of embeddings instead need only preserve within-item distance relationships. Note that $\boldsymbol{x}_i^j$ and $\boldsymbol{x}_i^{j'}$ are two different measurements of the same item, and $\boldsymbol{x}_{i'}^{j''}$ is an arbitrary measurement from a different item. If $\varphi(X) \triangleq \left\{\varphi(\boldsymbol{x}_i^j) : j \in [s]\right\}_{i\in[n]}$ is the set of points embedded by the within-item ordinal embedding φ, then the discriminability of $\varphi(X)$ is clearly the same as the discriminability of X. This is because the statement of a within-item ordinal embedding asserts that the relationship specified by discriminability holds *absolutely* (and therefore, it certainly also holds in probability). Note further that the class of embeddings which are local ordinal embeddings are a subset of the class of embeddings which are within-item ordinal embeddings.

Further, note that if φ were one-to-one, that the Bayes error is the same, which can be seen through a change of variables argument. These observations motivate the following corollary:

Corollary 3. *Suppose that* $\left\{(\boldsymbol{x}_i^j, y_i) : j \in [s]\right\}_{i\in[n]}$ *are stochastic measurements and class labels following the additive gaussian noise setting, described in Assumption 1.*

Let $\varphi : \mathcal{X} \to \mathcal{W}$ *be a within-item ordinal embedding which is also one-to-one, and denote* $\boldsymbol{w}_i^j = \varphi(\boldsymbol{x}_i^j)$*. There exists a decreasing function* $\gamma(\cdot)$ *of the discriminability* $D_w = D\left\{\boldsymbol{w}_i^j\right\}$ *where:*

$$L_\varphi^* \leq \gamma(D_w)$$

Proof. Denote $\gamma_x(\cdot)$ to be the decreasing function of $D_x = D\left\{\boldsymbol{x}_i^j\right\}$, which exists by Theorem (2), where:

$$L_x^* \leq \gamma_x(D_x)$$

Let L_x^* be the Bayes' error of $\left\{\boldsymbol{x}_i^j, y_i\right\}$. We note the following two facts:

1. The Bayes error $L_x^* = L_w^*$: Follows since φ is one-to-one.
2. $D_x = D_w$: Follows since φ is a local ordinal embedding.

Finally, using these two facts, note that:

$$L_w^* = L_x^* \leq \gamma_x(D_x) = \gamma_x(D_w)$$

So selecting the same function $\gamma = \gamma_x$ gives a function of the discriminability of $\left\{\boldsymbol{w}_i^j\right\}$ which upper bounds the Bayes' error of $\left\{(\boldsymbol{w}_i^j, y_i) : j \in [s]\right\}_{i\in[n]}$, L_w^*, as desired. ∎

Corollary 4. *Assume* (f_1, g_1) *and* (f_2, g_2) *are two analysis strategies, and suppose that* $D_{f_1,g_1} > D_{f_2,g_2}$*. Then the bound on the Bayes error for* (f_1, g_1) *is lower than the bound on the Bayes error on* (f_2, g_2)*.*

Proof. Direct application of Theorem 2, noting that $D_{f_1,g_1} > D_{f_2,g_2}$ implies that $\gamma(D_{f_1,g_1}) \leq \gamma(D_{f_2,g_2})$ since γ is decreasing in D. ∎

Consequently, under the described setting, the pipeline that achieves a higher `Discr` has a lower bound on the Bayes error than competing strategies, despite the fact that the task is unknown during data acquisition and analysis. Complementarily, note that if we were to instead consider the predictive accuracy $1 - L_{f,g}^*$, we can obtain a similar result to obtain a lower bound on the predictive accuracy via an increasing function of `Discr`. That is, in the context of the corollary, a more discriminable pipeline will tend to have a higher bound on the accuracy for an arbitrary predictive task.

References

1. Devroye L, Györfi L, Lugosi G. A probabilistic theory of pattern recognition. vol. 31. Springer Science & Business Media; 2013.
2. Paley R, Zygmund A. On some series of functions,(3). In: Mathematical Proceedings of the Cambridge Philosophical Society. vol. 28. Cambridge Univ Press; 1932. p. 190–205.
3. Devijver PA, Kittler J. Pattern recognition: A statistical approach. Prentice hall; 1982.
4. Terada Y, Luxburg U. Local ordinal embedding. 31st International Conference on Machine Learning, ICML 2014. 2014 Jan;3:2440–2458. Available from: https://www.researchgate.net/publication/288398272_Local_ordinal_embedding.
