# Supplementary material for: Eliminating accidental deviations to minimize generalization error and maximize replicability: Applications in connectomics and genomics
Source: PLoS Comput Biol. 2021 Sep 16;17(9):e1009279. doi: 10.1371/journal.pcbi.1009279 (PMC8500408; doi:10.1371/journal.pcbi.1009279)
Supplement: S4 Text — (PDF) [file pcbi.1009279.s004.pdf]

Supporting Information 4: Eliminating accidental deviations to minimize generalization error and maximize replicability: applications in connectomics and genomics

Eric W. Bridgeford[1], Shangsi Wang[1], Zeyi Wang[1], Ting Xu[3], Cameron Craddock[3], Jayanta Dey[1], Gregory Kiar[1], William Gray-Roncal[1], Carlo Colantuoni[1], Christopher Douville[1], Stephanie Noble[4], Carey E. Priebe[1], Brian Caffo[1], Michael Milham[3], Xi-Nian Zuo[2,5], Consortium for Reliability and Reproducibility, Joshua T. Vogelstein[1,6*]

S4 Simulations The following simulations were constructed, where $\sigma_{min}, \sigma_{max}$ are the variance ranges, and settings were run at 15 intervals in $[\sigma_{min}, \sigma_{max}]$ for 500 repetitions per setting. For a simulation setting with variance σ, the variance is reported as the normalized variance, $\bar{\sigma} = \frac{\sigma - \sigma_{min}}{\sigma_{max} - \sigma_{min}}$. Dimensionality is 2, the number of items is K, and the total number of measurements across all items is 128. Typically, i indicates the individual identifier, and j the measurement index. Notationally, in the below descriptions, we adopt the convention that $\boldsymbol{z}_i^j$ obeys the true distribution for a single observation j of item i, and $\boldsymbol{x}_i^j$ incorporates the controlled error term $\boldsymbol{\epsilon}_i^j$, which is the term which is varied the simulation. Further, each item features $\frac{n}{K}$ measurements.

Goodness of Fit Testing and Bayes Error

1. No Signal: $K = 2$ items, where the true distributions for class 1 and class 2 are the same.
   - $\boldsymbol{z}_i^j \overset{iid}{\sim} \mathcal{N}(\boldsymbol{0}, \boldsymbol{I})$, $i = 1, \ldots, 2$, $t = 1, \ldots, 64$. Note: $\boldsymbol{0} \in \mathbb{R}^2$ is $\boldsymbol{0}$, and likewise for $\boldsymbol{I}$
   - $\boldsymbol{\epsilon}_i^j \overset{iid}{\sim} \mathcal{N}\big(\boldsymbol{0}, \sigma^2 \boldsymbol{I}\big)$, $\sigma \in [0, 20]$
   - $\boldsymbol{x}_i^j = \boldsymbol{z}_i^j + \boldsymbol{\epsilon}_i^j \overset{iid}{\sim} \mathcal{N}\big(\boldsymbol{0}, (1 + \sigma^2)\boldsymbol{I}\big)$
2. Cross: $K = 2$ items, where the true distributions for class 1 and class 2 are orthogonal.
   - $\Sigma_1 = \begin{bmatrix} 2 & 0 \\ 0 & 0.1 \end{bmatrix}$, $\Sigma_2 = \begin{bmatrix} 0.1 & 0 \\ 0 & 2 \end{bmatrix}$
   - $\boldsymbol{z}_i^j \overset{iid}{\sim} \mathcal{N}(\boldsymbol{0}, \boldsymbol{\Sigma}_i)$, $i = 1, 2$
   - $\boldsymbol{\epsilon}_i^j \overset{iid}{\sim} \mathcal{N}\big(\boldsymbol{0}, \sigma^2 \boldsymbol{I}\big)$, $\sigma \in [0, 20]$
   - $\boldsymbol{x}_i^j = \boldsymbol{z}_i^j + \boldsymbol{\epsilon}_i^j$
3. Gaussian: $K = 16$ items, where the true distributions are each gaussian.
   - $\boldsymbol{\mu}_i \overset{iid}{\sim} \pi_1 \mathcal{N}(\boldsymbol{0}, 4\boldsymbol{I})$, $i = 1, \ldots, 16$
   - $\boldsymbol{\Sigma} = \begin{bmatrix} 1 & 0.1 \\ 0.1 & 1 \end{bmatrix}$
   - $\boldsymbol{z}_i^j \overset{iid}{\sim} \mathcal{N}(\boldsymbol{\mu}_i, \boldsymbol{\Sigma})$
   - $\boldsymbol{\epsilon}_i^j \overset{iid}{\sim} \mathcal{N}\big(\boldsymbol{0}, \sigma^2 \boldsymbol{I}\big)$, $\sigma \in [0, 20]$
   - $\boldsymbol{x}_i^j = \boldsymbol{z}_i^j + \boldsymbol{\epsilon}_i^j$
4. Ball/Circle: $K = 2$ items, where 1 item is uniformly distributed on the unit ball with gaussian error, and the second item is uniformly distributed on the unit sphere with gaussian error.
   - $\boldsymbol{z}_1^t \overset{iid}{\sim} \mathbb{B}(r = 1) + \mathcal{N}(\boldsymbol{0}, 0.1\boldsymbol{I})$ samples uniformly on unit ball of radius 2 with Gaussian error
   - $\boldsymbol{z}_2^t \overset{iid}{\sim} \mathbb{S}(r = 1.5) + \mathcal{N}(\boldsymbol{0}, 0.1\boldsymbol{I})$ samples uniformly on unit sphere of radius 2 with Gaussian error

[1] Johns Hopkins University, Baltimore, Maryland, USA, [2] Shanghai Jiaotong University, Shanghai, China [3] Child Mind Institute, New York, New York, USA [4] Yale University, New Haven, Connecticut, USA [5] Beijing Normal University, Beijing, China, Nanning Normal University, Nanning, China, University of Chinese Academy of Sciences, Beijing, China, [6] Progressive Learning, Baltimore, Maryland, USA. * jovo@jhu.edu.

- $\boldsymbol{\epsilon}_i^j \overset{iid}{\sim} \mathcal{N}\big(\mathbf{0}, \sigma^2 \boldsymbol{I}\big)$, $\sigma \in [0, 10]$
- $\boldsymbol{x}_i^j = \boldsymbol{z}_i^j + \boldsymbol{\epsilon}_i^j$

5. XOR: $K = 2$ items, where:
   - $\boldsymbol{z}_1^t = \begin{cases} \mathbf{0} & t \in 1, \dots, 32 \\ \mathbf{1} & t \in 33, \dots, 64 \end{cases}$
   - $\boldsymbol{z}_2^t = \begin{cases} [0, 1]' & t \in 1, \dots, 32 \\ [1, 0]' & t \in 33, \dots, 64 \end{cases}$
   - $\boldsymbol{\epsilon}_i^j \overset{iid}{\sim} \mathcal{N}\big(\mathbf{0}, \sigma^2 \boldsymbol{I}\big), \sigma \in [0, 0.8]$
   - $\boldsymbol{x}_i^j = \boldsymbol{z}_i^j + \boldsymbol{\epsilon}_i^j$

Bayes error was estimated by simulating $n = 10{,}000$ points according to the above simulation settings, and approximating the Bayes error through numerical integration. The classification labels for $K = 2$ simulations were consistent with the individual labels, and for the $K = 16$, the first class consists of the 8 distributions whose means were leftmost, and the rest of the distributions were the other class.

Comparison Testing Items are sampled with the same true distributions $\boldsymbol{z}_i^j$ as before, with the following augmentation:

$$\boldsymbol{x}_{i,k}^j = \begin{cases} \boldsymbol{z}_i^j & k = 1 \\ \boldsymbol{z}_i^j + \boldsymbol{\epsilon}_i^j & k = 2 \end{cases}$$

That is, the observed data $\boldsymbol{x}_{i,k}^j$ for item i, observation j, and sample $k \in [2]$ is such that the first sample is distributed according to the true item distribution, and the second sample is distributed according to the true item distribution with an added noise term, where $\boldsymbol{\epsilon}_i^j \overset{iid}{\sim} \mathcal{N}\big(\mathbf{0}, \sigma^2 \boldsymbol{I}\big)$:

1. No Signal: $K = 2$
   $\sigma \in [0, 10]$
2. Cross: $K = 2$
   $\sigma \in [0, 1]$
3. Gaussian: $K = 16$
   $\sigma \in [0, 1]$
4. Ball/Circle: $K = 2$
   $\sigma \in [0, 1]$
5. XOR: $K = 2$
   $\boldsymbol{x}_{i,k}^j = \begin{cases} \boldsymbol{z}_i^j + \boldsymbol{\tau}_i^j & k = 1 \\ \boldsymbol{z}_i^j + \boldsymbol{\tau}_i^j + \boldsymbol{\epsilon}_i^j & k = 1 \end{cases}$ where $\tau_i^j \overset{iid}{\sim} \mathcal{N}(\mathbf{0}, 0.1\boldsymbol{I})$
   $\sigma \in [0, 0.2]$

By construction, one would anticipate `Discr` of the first sample to exceed that of the second sample, as the second sample has additional error. Therefore, the natural hypothesis is:

$$H_0 : D^{(1)} = D^{(2)}, \qquad H_A : D^{(1)} > D^{(2)}$$
