# Supplementary material for: Eliminating accidental deviations to minimize generalization error and maximize replicability: Applications in connectomics and genomics
Source: PLoS Comput Biol. 2021 Sep 16;17(9):e1009279. doi: 10.1371/journal.pcbi.1009279 (PMC8500408; doi:10.1371/journal.pcbi.1009279)
Supplement: S5 Text — (PDF) [file pcbi.1009279.s005.pdf]

Supporting Information 5: Eliminating accidental deviations to minimize generalization error and maximize replicability: applications in connectomics and genomics

Eric W. Bridgeford[1], Shangsi Wang[1], Zeyi Wang[1], Ting Xu[3], Cameron Craddock[3], Jayanta Dey[1], Gregory Kiar[1], William Gray-Roncal[1], Carlo Colantuoni[1], Christopher Douville[1], Stephanie Noble[4], Carey E. Priebe[1], Brian Caffo[1], Michael Milham[3], Xi-Nian Zuo[2,5], Consortium for Reliability and Reproducibility, Joshua T. Vogelstein[1,6*]

S5 Hypothesis Testing

Goodness of Fit Test Recall the goodness of fit test, shown in Equation (1). We approximate the distribution of $\hat{S}$ under the null through a permutation approach. The item labels of our N samples are first permutated randomly, and $\hat{S}_{0,N}$ is computed each time given the observed data $\boldsymbol{X}$ and the permuted labels. For a level α significance test, we compare $\hat{S}$ to the $(1-\alpha)$ quantile $\mathcal{Q}_{1-\alpha}$ of the empirical null distribution $\hat{D}_{0,N}$, and reject the null hypothesis if $\hat{D}_N < \mathcal{Q}_{1-\alpha}$. This approach provides a consistent and valid test under general assumptions.

Note that the permutation-based approach requires r computations of the sample `Discr`. The total computational complexity is then $\mathcal{O}\big(N^2 \max(p, rs)\big)$. This approach is only linear in the number of desired repetitions, and therefore is sensible for most settings in which the sample `Discr` can itself be computed. Moreover, we can greatly speed this computation up through parallelization. With T cores, the computational complexity is instead $\mathcal{O}\big(N^2 \max\big(p, \frac{r}{T}s\big)\big)$, as shown in Algorithm 1. We extend this goodness of fit test to both `PICC` and `I2C2` to provide a robust p-value associated with both statistics of interest. Note that the permutation approach can be generalized to any statistic quantifying replicability based on repeated measurements.

[1] Johns Hopkins University, Baltimore, Maryland, USA, [2] Shanghai Jiaotong University, Shanghai, China [3] Child Mind Institute, New York, New York, USA [4] Yale University, New Haven, Connecticut, USA [5] Beijing Normal University, Beijing, China, Nanning Normal University, Nanning, China, University of Chinese Academy of Sciences, Beijing, China, [6] Progressive Learning, Baltimore, Maryland, USA. * jovo@jhu.edu.

Algorithm 1 `Discr` **Goodness of Fit Test**. Our implementation of the permutation test for the goodness of fit test of the hypothesis given in Equation (1) requires $\mathcal{O}\big(N^2 \max\big(p, \frac{r}{T}s\big)\big)$ time, where r is the number of permutations and T is the number of cores available for the permutation test. The `Shuffle` function is the function which rearranges all of the data within the dataset, without regard to item nor measurement index. The output provides a new measurement index for each item i and measurement j.

Require: (1) $\left\{\boldsymbol{x}_i^j\right\}_{j\in[J_i],i\in[n]}$ n items of data, each featuring J_i measurements.
(2) r an integer for the number of permutations.

Ensure: $p \in [0,1]$ the p-value associated with the test.

```
function p = GoodnessOfFitTest({x_i^j}_{j∈[J_i],i∈[n]}, r)
    d_a = Discr{x_i^j}_{j∈[J_i],i∈[n]}                    ▷ compute observed sample Discr
▷ Note that this for-loop can be parallelized over T cores, as the loops are independent
    for i in 1,...,r do
        π = Shuffle(n, {J_i}_{i=1}^n)                     ▷ a random shuffling of the measurements
        d_i = Discr{x_{π(i,j)}}_{j∈[J_i],i∈[n]}            ▷ Compute Discr with random order of sample ids
    end for
    p = 1/(r+1) (Σ_{i=1}^r 𝕀_{d_a ≥ d_i} + 1)  ▷ p-value is fraction of times observed is more extreme than under null
    return p
end function
```

Comparison Test We implement Comparison testing using a permutation approach, similar to the goodness of fit test. First, compute the observed difference in `Discr` between two design choices. The null distribution of the difference in `Discr` is constructed by first taking random convex combinations of the observed data from each of the two methods choices (the "randomly combined datasets"). `Discr` is computed for each of the two randomly combined datasets for each permutation. Finally, for each permutation, the all pairs of observed differences in `Discr` is computed. Finally, the observed statistic is compared with the differences under the null of the randomly combined datasets. The p-value is the fraction of times that the observed statistic is more extreme than the null. Note that we can use this approach for both one and two-tailed hypotheses for an experimental design having higher `Discr`, lower `Discr`, and equal `Discr` relative a second approach; we implement all three in the software implementation of the comparison test. The Algorithm for the comparison test is shown in Algorithm 2, with the alternative hypothesis as specified in Equation (2). The computational complexity is then $\mathcal{O}\big(\frac{r}{T}N^2\max(p, \max_i(s_i))\big)$. Note that for each permutation, the limiting step is the computation of the `Discr` in $\mathcal{O}\big(N^2\max(p, s)\big)$. This is then offset through parallelization over T cores in the implementation. We extend this comparison test to all competing approaches to provide a robust p-value associated with both statistics of interest, for similar reasons to the above. Again, this permutation approach can be generalized to any statistic quantifying replicability based on repeated measurements.

Algorithm 2 `Discr` **Discriminability Comparison Test**.Our implementation of the permutation test for the hypothesis given in Equation (2) requires $\mathcal{O}\big(\frac{r}{T}N^2 \max(p,s)\big)$ time, where r is the number of permutations and T is the number of cores available for the permutation test. Above, the only alternative considered is that $H_A : D^{(1)} > D^{(2)}$; our code-based implementation provides strategies for $H_A : D^{(1)} < D^{(2)}$ and $H_A : D^{(1)} = D^{(2)}$ as well.

Require: (1) $\left\{\boldsymbol{x}_i^j\right\}_{j\in[J_i],i\in[n]}$ n items of data, each featuring J_i measurements, from the first sample.
(2) $\left\{\boldsymbol{z}_i^j\right\}_{j\in[J_i],i\in[n]}$ n the observed data, from the second sample.
(3) r an integer for the number of permutations.

Ensure: $p \in [0,1]$ the p-value associated with the test.

function $p =$ComparisonTest($\{\boldsymbol{x}_i^j\}_{j\in[J_i],i\in[n]}, \{\boldsymbol{z}_i^j\}_{j\in[J_i],i\in[n]}, r$)
  $\hat{D}^{(1)} = \texttt{Discr}\left\{\boldsymbol{x}_i^j\right\}_{j\in[J_i],i\in[n]}$ ▷ The `Discr` of the first sample.
  $\hat{D}^{(2)} = \texttt{Discr}\left\{\boldsymbol{z}_i^j\right\}_{j\in[J_i],i\in[n]}$ ▷ The `Discr` of the second sample.
  $d_a = \hat{D}^{(1)} - \hat{D}^{(2)}$ ▷ The observed difference in `Discr` between samples 1 and 2.
  ▷ The for-loop below can be parallelized over T cores, as each loop is an independent
  for i in $1 : r$ **do**
    ▷ Generate a synthetic null dataset for each of the 2 samples, using a convex combination of the elements of each sample
    for k in $1 : 2$ **do**
      $\pi = \texttt{Shuffle}(n, \{J_i\}_{i=1}^n)$ ▷ a random shuffle of the measurements
      $\psi = \texttt{Shuffle}(n, \{J_i\}_{i=1}^n)$
      $\lambda_i^j \overset{iid}{\sim} \mathrm{Unif}(0,1)$ ▷ for $j = 1, \dots, n$, where $\boldsymbol{\Lambda} = (\lambda_j)_{j=1}^n$
      $\boldsymbol{u}_i^j = \lambda_i^j \boldsymbol{x}_{\pi(i,j)} + (1-\lambda_i^j)\boldsymbol{z}_{\psi(i,j)}$ ▷ Convex combination of random elements from each sample
      $d_i^{(k)} = \texttt{Discr}\left\{\boldsymbol{u}_i^j\right\}_{j\in[J_i],i\in[n]}$ ▷ Compute `Discr` of the convexly combined elements
    end for
  end for
  ▷ Compute all pairs differences in `Discr` using the convexly-combined samples
  for i in $1, \dots, r-1$ **do**
    for j in $i+1, \dots, r$ **do**
      $d_n \leftarrow c\Big(d_n, d_{n,i}^{(1)} - d_{n,j}^{(2)}, d_{n,j}^{(2)} - d_{n,i}^{(1)}\Big)$ ▷ Null distribution of the difference
    end for
  end for
  ▷ p-value is fraction of times that observed `Discr` is more extreme than synthetic datasets
  $p = \frac{2}{r(r-1)+1}\left(\sum_{i=1}^{|d_n|} \mathbb{I}_{\{d_a \leq d_{n,i}\}} + 1\right)$
  return p
end function
