# Supplementary material for: Eliminating accidental deviations to minimize generalization error and maximize replicability: Applications in connectomics and genomics
Source: PLoS Comput Biol. 2021 Sep 16;17(9):e1009279. doi: 10.1371/journal.pcbi.1009279 (PMC8500408; doi:10.1371/journal.pcbi.1009279)
Supplement: S6 Text — (PDF) [file pcbi.1009279.s006.pdf]

Supporting Information 6: Eliminating accidental deviations to minimize generalization error and maximize replicability: applications in connectomics and genomics

Eric W. Bridgeford[1], Shangsi Wang[1], Zeyi Wang[1], Ting Xu[3], Cameron Craddock[3], Jayanta Dey[1], Gregory Kiar[1], William Gray-Roncal[1], Carlo Colantuoni[1], Christopher Douville[1], Stephanie Noble[4], Carey E. Priebe[1], Brian Caffo[1], Michael Milham[3], Xi-Nian Zuo[2,5], Consortium for Reliability and Reproducibility, Joshua T. Vogelstein[1,6*]

S6 Connectomics Application

Data Acquisition and Analysis

fMRI Analysis Pipelines The fMRI connectomes were acquired as follows. Motion correction is performed via `mcflirt` to estimate the 6 motion parameters (x, y, z translation and rotations). Registration is performed by first performing a cross-modality registration from the functional to the anatomical MRI using `flirt-bbr`, followed by registration to the anatomical template using either (1) FSL-`fnirt` or (2) ANTs-`SyN`, two techniques for non-linear registration. Frequency filtering was performed by either (1) not frequency filtering, or (2) bandpass filtering signal outside of the $[.01, .1]$ Hz range. Volumes were either (1) not scrubbed, or (2) scrubbed if motion exceeded 0.5 mm, in which case the preceding volume and succeeding two volumes were removed. Global signal regression was either (1) not performed, or (2) performed by removing the global mean signal across all voxels in the functional timeseries. Moreover, across all analysis pipelines, the top 5 principal components (`compcor`), Friston 24 parameters, and a quadratic polynomial were fit and regressed from the functional timeseries. Finally, the voxelwise timeseries were spatially downsampled using (1) the CC200 parcellation, (2) the AAL parcellation, (3) the Harvard-Oxford parcellation, or (4) the Desikan-Killany parcellation. Graphs were estimated by (1) computing the rank of the non-zero raw absolute correlations (zero-weight edges given a value of 0), (2) log-transforming the raw absolute correlations (the minimum value of the graph is down-scaled by a factor of 100 and then added to each edge to eliminate taking $\log$ of zero-weight edges), or (3) computing the raw absolute correlation between pairs of regions of interest in each parcellation. No mean centering was performed for functional connectivity estimates. Specific data analysis instructions for deployment in `AWS` can be found in the https://neurodata.io/m2g. All data analysis was performed in the `AWS` cloud using `CPAC` version `3.9.2` [1]. All parcellations are available in `neuroparc` human brain atlases [2].

dMRI Analysis Pipelines The dMRI connectomes were acquired as follows. The dMRI scans were corrected for eddy currents using FSL's `eddy-correct` [3]. FSL's "standard" linear registration pipeline was used to register the sMRI and dMRI images to the MNI152 atlas [3–6]. A tensor model is fit using DiPy [7] to obtain an estimated tensor at each voxel. A deterministic tractography algorithm is applied using DiPy's EuDX [7, 8] to obtain streamlines, which indicate the voxels connected by an axonal fiber tract. Graphs are formed by contracting voxels into graph vertices depending on spatial [9], anatomical [10–13], or functional [14–17] similarity. Given a parcellation with vertices V and a corresponding mapping $P(v_i)$ indicating the voxels within a region i, we contract our fiber streamlines as follows. $w(v_i, v_j) = \sum_{u \in P(v_i)} \sum_{w \in P(v_j)} \mathbb{I}\{F_{u,w}\}$ where $F_{u,w}$ is true if a fiber tract exists between voxels u and w, and false if there is no fiber tract between voxels u and w. The specific parcellations leveraged are detailed in **(author?)** [18], consisting of parcellations defined in the MNI152 space [10–17]. The

[1] Johns Hopkins University, Baltimore, Maryland, USA, [2] Shanghai Jiaotong University, Shanghai, China [3] Child Mind Institute, New York, New York, USA [4] Yale University, New Haven, Connecticut, USA [5] Beijing Normal University, Beijing, China, Nanning Normal University, Nanning, China, University of Chinese Academy of Sciences, Beijing, China, [6] Progressive Learning, Baltimore, Maryland, USA. * jovo@jhu.edu.

graphs are then re-weighted using the afforementioned weighting schemes described in $\text{fMRI Analysis Pipelines}$ Supplementary Information ; namely, the raw, ranked, and $\log$ edge-weights. All parcellations are available in `neuroparc` human brain atlases [2].

PCR RealSeqS Cancer Genomics Pipeline The RealSeqS samples were acquired as follows. PCR was performed in 25 μL reactions containing 7.25 μL of water, 0.125 μL of each primer, 12.5 μL of NEBNext Ultra II Q5 Master Mix (New England Biolabs cat # M0544S), and 5 μL of DNA. The cycling conditions were: one cycle of 98°C for 120 s, then 15 cycles of 98°C for 10 s, 57°C for 120 s, and 72°C for 120 s. Each plasma DNA sample was assessed in eight independent reactions, and the amount of DNA per reaction varied from 0.1 μg to 0.25 μg. A second round of PCR was then performed to add dual indexes (barcodes) to each PCR product prior to sequencing. The second round of PCR was performed in 25 μL reactions containing 7.25 μL of water, 0.125 μL of each primer, 12.5 μL of NEBNext Ultra II Q5 Master Mix (New England Biolabs cat # M0544S), and 5 uL of DNA containing 5% of the PCR product from the first round. The cycling conditions were: one cycle of 98Âřč for 120 s, then 15 cycles of 98°C for 10 s, 65°C for 15 s, and 72°C for 120 s. Amplification products from the second round were purified with AMPure XP beads (Beckman cat # a63880), as per the manufacturer's instructions, prior to sequencing. As noted above, each sample was amplified in eight independent PCRs in the first round. Each of the eight independent PCRs was then re-amplified using index primers in the second PCR round. `Bowite2` was then used to align reads to the human reference genome assembly GRC37 [19] for each well. After alignment to $\sim 750,000$ amplicons, the wells were downsampled into non-overlapping windows of 5×10^4 bases, 5×10^5 bases, 5×10^6 bases, or to the individual chromosome level (the resolution of the data).

Effect Size Investigation In this investigation, we are interested in learning how maximization based on the observed notion of replicability correlates with real performance on a downstream inference task. Recalling Corollary 4 from S3, we explore the implications of this corollary in a large neuroimaging dataset provided by the Consortium for Reliability and Reproducibility [20], and demonstrate that selection of the experimental design via `Discr`, in fact, facilitates improved downstream inference on both a regression and classification task. We further extend this to two separate genomics datasets investigating classification tasks, and again demonstrate that selection of experimental design via `Discr` improves downstream inference. This provides strong motivation for leveraging the `Discr` for experimental design.

Ideally, for a particular summary reference statistic, a high value will generally correlate with a positive effect size. For datasets $i = 1, \ldots, M$ where M is the total number of datasets, an analysis strategy $j = 1, \ldots, 192$ for 192 total analysis strategies, and $k = 1, \ldots, 3$ are our summary reference statistics of interest (`Discr`, `PICC`, `Fingerprint`, `I2C2`, `Kernel`), we fit the standard linear regression model $Y = \beta X + \epsilon$, where we model the effect size Y estimated by `DCorr` [21] via a linear relationship with X, the observed reference statistic for approach k, with coefficient β. Note that the interpretation of β is the expected change in the effect size Y due to a single unit change in the observed reference statistic X. Both Y and X are uniformly normalized across all strategies within a single dataset to facilitate intuitive comparison across methods. For each reference statistic k, we pose the following hypothesis:

$$H_0 : \beta = 0; \quad H_A : \beta > 0$$

Acceptance of the alternative hypothesis would have the interpretation that an increase in the observed reference statistic X would tend to correspond to an increase in the observed effect size Y, and the relevant test is the one-way Z-test. To robustify against model assumptions, we use robust standard errors [22]. Acceptance of the alternative hypothesis against the null provides evidence that an increase in the sample statistic corresponds to an increase in the observed effect size, where the responses (age, sex, cancer status) were not considered at the time the data were analyzed nor when the reference statistics computed. This provides evidence that the statistic is informative for experimental design

Dataset	Manuf.	Model	TE (ms)	TR (ms)	STC	#Timepts	#Sub	#Ses	#Scans	TRT (days)	Discr
KKI2009	Philips	Achieva	30	2000	seq.	210	21	2	42	<1	0.93
NKI24	Siemens	TrioTim	30	645	inter.	900	24	2	47	<14	0.98
BNU1	Siemens	TrioTim	30	2000	inter.	200	50	2	100	42	0.97
BNU2	Siemens	TrioTim	30	variable	inter.	variable	50	2	100	103	0.92
DC1	Philips	NA	35	2500	inter.	120	114	4	244	?	0.95
HNU1	GE	MR750	30	2000	inter.	300	30	10	300	3	0.98
IACAS	GE	Signa	30	2000	inter.	240	28	3	59	42	0.83
IBATRT	Siemens	TrioTim	30	1750	seq.	220	36	2	50	None	0.95
IPCAS	NA	NA	NA	NA	NA	NA	78	2	156	–	0.99
IPCAS1	Siemens	TrioTim	30	2000	inter.	205	30	2	60	7	1.00
IPCAS2	Siemens	TrioTim	30	2500	inter.	212	35	2	70	30	0.98
IPCAS5	Siemens	TrioTim	30	2000	inter.	170	22	2	44	>10 (min)	0.96
IPCAS6	Siemens	TrioTim	30	2500	inter.	242	2	15	30	3 (hrs)	1.00
IPCAS8	Siemens	TrioTim	30	2000	inter.	240	13	2	26	>1 (years)	0.96
JHNU	Siemens	TrioTim	30	2000	inter.	250	30	2	60	NA	0.96
LMU3	Siemens	TrioTim	30	3000	inter.	120	25	2	50	NA	0.93
MRN1	NA	NA	NA	NA	NA	NA	53	2	88	120	0.94
NYU1	Siemens	Allegra	25	2000	NaN	197	25	3	75	5-11 (mo)*	0.98
NYU2	Siemens	Allegra	15	2000	inter.	180	187	3	252	<1 (hrs)	0.96
SWU1	Siemens	TrioTim	30	2000	inter.	240	20	3	59	NA	0.97
SWU2	Siemens	TrioTim	30	2000	inter.	300	27	2	54	NA	0.96
SWU3	Siemens	TrioTim	30	2000	inter.	242	24	2	48	NA	0.98
SWU4	Siemens	TrioTim	30	2000	inter.	242	235	2	467	1 (yrs)	0.97
UM	Siemens	TrioTim	30	2000	seq.	150	80	2	160	NA	0.99
UPSM1	Siemens	TrioTim	29	1500	seq.	200	100	3	230	473 - 1434	0.89
Utah1	Siemens	TrioTim	28	2000	inter.	240	26	2	52	>2 (yrs)	0.92
UWM	GE	MR750	25	2600	inter.	231	25	2	50	NA	0.96
XHCUMS	Siemens	TrioTim	30	3000	inter.	124	24	5	120	180	0.91

S6 Table 1. fMRI Dataset Descriptions. In the above table, STC corresponds to slice timing correction. Rows with NA entries do not have available metadata associated with the scanning protocol. The column TRT indicates the follow up time for retest. A value of None indicates that the scans were back to back. The sample Discr corresponds to the Discr of the best performing pipeline overall, FNNNCP. *The test-retest structure for NYU1 was 5 - 11 months between sessions 1 and 2, and $30 - 45$ minutes between sessions 2 and 3.

within the context of this investigation. Model fitting for this investigation is conducted using the lm package in the R programming language [23].

Human Brain Imaging Dataset Descriptions

Dataset	Manuf.	Model	TE (ms)	TR (ms)	#Dir	bval $\frac{s}{mm^2}$	#Sub	#Ses	#Scans	TRT (days)	Discr
BNU1	Siemens	TrioTim	89	8000	30	1000	57	2	113	42	1.00
HNU1	GE	MR750	Min	8600	33	1000	30	10	300	3	0.99
KKI2009	Philips	NA	32	6281	65	700	21	2	42	<1	1.00
NKI24	Siemens	TrioTim	95	2400	137	1500	20	2	40	<14	1.00
SWU4	Siemens	TrioTim	NaN	NaN	93	1000	227	2	454	1 (yrs)	0.88

S6 Table 2. dMRI Dataset Descriptions. In the above table, #Dir corresponds to the number of diffusion directions. Rows with NA entries do not have available metadata associated with the scanning protocol. The sample Discr corresponds to the Discr of the pipeline with the CPAC200 parcellation and the log-transformed edges.

Useful Data Links All relevant analysis scripts and data for figure reproduction in this manuscript made publicly available, and can be found at https://neurodata.io/mgc.

References

1. Craddock C, Sikka S, Cheung B, Khanuja R, Ghosh SS, Yan C, et al. Towards Automated Analysis of Connectomes: The Configurable Pipeline for the Analysis of Connectomes (C-PAC). Frontiers in Neuroimformatics. 2013 Jul;.
2. Lawrence RM, Bridgeford EW, Myers PE, Arvapalli GC, Ramachandran SC, Pisner DA, et al. Standardizing human brain parcellations. Sci Data. 2021 Mar;8(78):1–9.
3. Smith SM, et al. Advances in functional and structural MR image analysis and implementation as FSL. NeuroImage. 2004 jan;23 Suppl 1:S208–19. Available from: http://www.ncbi.nlm.nih.gov/pubmed/15501092.
4. Woolrich MW, et al. Bayesian analysis of neuroimaging data in FSL. NeuroImage. 2009 mar;45(1 Suppl):S173–86. Available from: http://www.sciencedirect.com/science/article/pii/S1053811908012044.
5. Jenkinson M, et al. FSL. NeuroImage. 2012 aug;62(2):782–90. Available from: http://www.ncbi.nlm.nih.gov/pubmed/21979382.
6. Mazziotta J, et al. A four-dimensional probabilistic atlas of the human brain. Journal of the American Medical Informatics Association. 2001;8(5):401–430.
7. Garyfallidis E, Brett M, Amirbekian B, Rokem A, Van Der Walt S, Descoteaux M, et al. Dipy, a library for the analysis of diffusion MRI data. Frontiers in neuroinformatics. 2014;8:8.
8. Garyfallidis E, Brett M, Correia MM, Williams GB, Nimmo-Smith I. Quickbundles, a method for tractography simplification. Frontiers in neuroscience. 2012;6:175.
9. Mhembere D, Roncal WG, Sussman D, Priebe CE, Jung R, Ryman S, et al. Computing scalable multivariate glocal invariants of large (brain-) graphs. In: Global Conference on Signal and Information Processing (GlobalSIP), 2013 IEEE. IEEE; 2013. p. 297–300.
10. Tzourio-Mazoyer N, et al. Automated anatomical labeling of activations in SPM using a macroscopic anatomical parcellation of the MNI MRI single-subject brain. Neuroimage. 2002;15(1):273–289.
11. Oishi K, et al. MRI atlas of human white matter. Academic Press; 2010.
12. Makris N, Goldstein JM, Kennedy D, Hodge SM, Caviness VS, Faraone SV, et al. Decreased volume of left and total anterior insular lobule in schizophrenia. Schizophrenia research. 2006;83(2):155–171.
13. Lancaster J. The Talairach Daemon, a database server for Talairach atlas labels. NeuroImage. 1997;.
14. Craddock RC, Jbabdi S, Yan CG, Vogelstein JT, Castellanos FX, Di Martino A, et al. Imaging human connectomes at the macroscale. Nat Methods. 2013 Jun;10(6):524–539. Available from: http://dx.doi.org/10.1038/nmeth.2482.
15. Sripada CS, et al. Lag in maturation of the brain's intrinsic functional architecture in attention-deficit/hyperactivity disorder. Proceedings of the National Academy of Sciences. 2014;111(39):14259–14264.
16. Kessler D, et al. Modality-spanning deficits in attention-deficit/hyperactivity disorder in functional networks, gray matter, and white matter. The Journal of Neuroscience. 2014;34(50):16555–16566.
17. Desikan RS, et al. An automated labeling system for subdividing the human cerebral cortex on MRI scans into gyral based regions of interest. NeuroImage. 2006;.
18. Kiar G, Bridgeford E, Roncal WG, (CoRR) CfR, Reproducibliity, Chandrashekhar V, et al. A High-Throughput Pipeline Identifies Robust Connectomes But Troublesome Variability. bioRxiv. 2018 Apr;p. 188706. Available from: https://www.biorxiv.org/content/early/2018/04/24/188706.
19. Langmead B, Salzberg SL. Fast gapped-read alignment with Bowtie 2. Nat Methods. 2012 Mar;9(4):357–359.
20. Zuo XN, Anderson JS, Bellec P, Birn RM, Biswal BB, Blautzik J, et al. An open science resource for establishing reliability and reproducibility in functional connectomics. Scientific data.

2014;1:140049.
21. Shen C, Vogelstein JT. Decision Forests Induce Characteristic Kernels. arXiv. 2018 Nov;Available from: http://arxiv.org/abs/1812.00029.
22. Zeileis A. Object-oriented Computation of Sandwich Estimators. Journal of Statistical Software, Articles. 2006;16(9):1–16.
23. R Core Team. R: A Language and Environment for Statistical Computing. Vienna, Austria; 2013. ISBN 3-900051-07-0. Available from: http://www.R-project.org/.
