# Supplementary material for: Eliminating accidental deviations to minimize generalization error and maximize replicability: Applications in connectomics and genomics
Source: PLoS Comput Biol. 2021 Sep 16;17(9):e1009279. doi: 10.1371/journal.pcbi.1009279 (PMC8500408; doi:10.1371/journal.pcbi.1009279)
Supplement: S7 Text — (PDF) [file pcbi.1009279.s007.pdf]

Supporting Information 7: Eliminating accidental deviations to minimize generalization error and maximize replicability: applications in connectomics and genomics

Eric W. Bridgeford[1], Shangsi Wang[1], Zeyi Wang[1], Ting Xu[3], Cameron Craddock[3], Jayanta Dey[1], Gregory Kiar[1], William Gray-Roncal[1], Carlo Colantuoni[1], Christopher Douville[1], Stephanie Noble[4], Carey E. Priebe[1], Brian Caffo[1], Michael Milham[3], Xi-Nian Zuo[2,5], Consortium for Reliability and Reproducibility, Joshua T. Vogelstein[1,6*]

S7 Discriminability Decomposition Consider data which is observed as the pairs (x_i^k, y_i), where $i = 1, \dots, n$ indexes subjects, and $k = 1, \dots, s$ indexes sessions. We suppose that x_i^k represents a measurement of interest, and y_i represents a subject-specific categorical class of interest (such as a natively categorical covariate such as sex, or a natively numeric covariate such as age which can be coerced to categorical; e.g., using age quintiles or deciles). Interestingly, the discriminability can be separated into the within-class and between-class contributions on the basis of y_i.

Discriminability Decomposition Using the definition of the discriminability:

$$D \triangleq \mathbb{P}\Big(\delta(x_i, x_i^{k'}) < \delta(x_i^k, x_j^{k''})\Big)$$
$$= \mathbb{P}\Big(\delta(x_i, x_i^{k'}) < \delta(x_i^k, x_j^{k''}) \big| y_i = y_j\Big)\mathbb{P}(y_i = y_j) + \mathbb{P}\Big(\delta(x_i, x_i^{k'}) < \delta(x_i^k, x_j^{k''}) \big| y_i \neq y_j\Big)\mathbb{P}(y_i \neq y_j)$$

Which follows by the law of total probability. Let $\omega = \mathbb{P}(y_i = y_j)$ be the probability of a pair of elements being from he same class, $W = \mathbb{P}\Big(\delta(x_i, x_i^{k'}) < \delta(x_i^k, x_j^{k''}) \big| y_i = y_j\Big)$ be the discriminabilty of items which are from the same class (the *within-class* discriminability). The within-class discriminability represents the discriminability computed between pairs of items which are from the same-class.

Further, let $\beta = \mathbb{P}(y_i \neq y_j)$ the probability that two items are from different classes, and $B = \mathbb{P}\Big(\delta(x_i, x_i^{k'}) < \delta(x_i^k, x_j^{k''}) \big| y_i \neq y_j\Big)$ be the discriminability of items which are from different classes (the *between-class* discriminability). The between-class discriminability represents the discriminability computed between pairs of items which are from different classes.

Then:

$$D = \omega W + \beta B$$

Showing that discriminability can be decomposed as a weighted sum of the within and between-class discriminabilities.

[1] Johns Hopkins University, Baltimore, Maryland, USA, [2] Shanghai Jiaotong University, Shanghai, China [3] Child Mind Institute, New York, New York, USA [4] Yale University, New Haven, Connecticut, USA [5] Beijing Normal University, Beijing, China, Nanning Normal University, Nanning, China, University of Chinese Academy of Sciences, Beijing, China, [6] Progressive Learning, Baltimore, Maryland, USA. * jovo@jhu.edu.
