# Supplementary material for: Eliminating accidental deviations to minimize generalization error and maximize replicability: Applications in connectomics and genomics
Source: PLoS Comput Biol. 2021 Sep 16;17(9):e1009279. doi: 10.1371/journal.pcbi.1009279 (PMC8500408; doi:10.1371/journal.pcbi.1009279)
Supplement: Attachment — Submitted filename: Strother_ Review_PCOMPBIOL-D-21-00089.pdf [file pcbi.1009279.s008.pdf]

I enjoyed reading this manuscript, which introduces a number of important ideas and associated practical analytic techniques to the experimental test-retest literature, and then illustrates their use in an impressive analysis of processing choices in big data settings for neuroimaging and genomics. To achieve this the authors introduce a non-parametric comparison of distance ranks under some metric--Euclidean for the purposes of this manuscript--comparing between- versus within-item pairwise distances as a possible way to address the scientific reproducibility/replication challenge that has been highlighted in many fields of late. The authors calculate this metric Discr as the fraction of all possible pairwise distances in the sample for which the within-item distances (e.g., within subject) are smaller than between-item distances. As such it is an interesting, non-trivial, nonparametric extension of the classical multi-class (Fisher) discriminant that maximises between versus within variance/covariance structure and it thereby allows for a similar and intuitive interpretation of discriminable items, but in a non-parametric and potentially non-Gaussian setting, i.e., Discr attains the maximum value of 1 when all items are discriminable given relatively small within-item and large between-item distances. They also explore possible nonparametric, non-Gaussian kernel generalisations of the classical multi-class discriminant and show that over their simulations this does not perform as well as Discr. because of the small within samples and large between samples typical of test-retest experiments. I believe this is an important insight that considerably strengthens their scientific contribution.

They could spend a little more time discussing the practical pros and cons of their approach as it is not obvious to me that with item=subject in neuroimaging the non-stationarity of human brain signals over time necessarily implies that minimising within-subject pattern distances only up to the point that they are less than between-subject differences is a sufficient metric for most criterion validity. See my comments re. saturation of Discr below, although I recognise that the results in Fig. 5 provide a strong case for the utility of Discr wrt the particular case of criterion validity for subject-dependent covariates.

In addition to k-class kernel methods the authors compare the performance of their Discr measure to several existing measures of data reproducibility, namely their multivariate intra-class correlation coefficient (I2C2) and a fingerprinting metric introduced in the functional neuroimaging literature. They do an good job of arguing the benefits of Discr using simulations for which I found the simulations and figures very informative. They also show that in real data, Discr appears to track with "criterion validity" such as ageand sex in functional neuroimaging, although their claim that this is the "the only multivariate measure of reliability that has been explicitly related to criterion validity" is incorrect (see below). Nevertheless, their analysis of fMRI and DTI pipeline choices is impressive and seems relatively comprehensive given the particular preprocessing and analysis choices compared. I am less familiar with the genomic framework analysed but it is a strength of the manuscript that they demonstrate the utility of Discr in two separate problem domains.

At places in the manuscript reliability seems to be defined as computation reproducibility and is used interchangeably with general reproducibility. For example on p. 1: " Engineering and operations research have been concerned with reliability for a long time, as they require that their products are reliable under various conditions" and "A different strategy has been to quantify the reproducibility, or reliability, of ones' data ...". It would be helpful to have these terms very clearly defined based on the 2019 report of the National Academies of Sciences, Engineering and Medicine, "Reproducibility and Reliability in Science" (http://nap.edu/25303), and then used in the context of these clear definitions. In addition, the terms repeatability, stability and robustness are also mentioned as being closely related but again no distinguishing definitions are provided.

P2, "... many studies have been devoted to quantifying the reproducibility of different univariate properties of the data [14–17]". This is a highly selective set of mainly recent references so at a minimum needs to be proceeded by e.g., indicating that a much older and larger literature exists. In particular, there are multiple publications that have considered issues well beyond the basic ICCs that have come to dominate the more recent reliability, test-retest literature, e.g., https://pubmed.ncbi.nlm.nih.gov/10408770/ from 1999. In this sense the improvement of Discr over ICC or even I2C2 is clear but does not constitute all of the neuroimaging literature on test-retest performance.

P2, "Specifically, we advocate for explicitly and specifically designing experiments to ensure that they provide highly reproducible data, rather than hoping that they do and performing post-hoc checks after collecting the data." This sentence seems to imply the techniques to be discussed will require no knowledge of, or post-hoc measurements derived from the experimental data, but Discr can only be calculated given experimental data. Please be clearer about exactly what post-hoc checks you expect to avoid and how these will be avoided? Your bolded sentence seems to promise more than your technique delivers. In fact you note the caveat that no reproducibility metric can guarantee general criterion avlidity and this rather important limitation should be reflected in other statements throughout the manuscript.

P2. Please provide full references with links for ref. 25 and 26.

P2. The authors claim that finally and most uniquely, " Discr is the only non-parametric multivariate measure of test-retest reliability with formal theoretical guarantees of convergence and upper bounds on subsequent inference performance." I agree with the authors that this is a potentially important feature to have and congratulate the authors on their proof. But this bound is conflated with their statement that "(Discr) it is nonparametric, meaning that its validity and interpretation do not depend on any parametric assumptions, such as Gaussianity." On p. 5 the authors state their "Theorem 1. Under the multivariate Gaussian mixture model plus additive Gaussian noise setting, Discr provides a lower bound on the predictive accuracy of a subsequent classification task. Consequently, a strategy with a higher Discr provably provides a higher bound on predictive accuracy than a strategy with a lower Discr." It seems that their bound only formally exists for multivariate Gaussian models so that they should not claim the existence of the bound independent of "... any parametric assumptions, such as Gaussianity." Please modify these statements to include the appropriate caveats.

P3. List of contributions 1-6. In general I am OK with these except where I believe they overstate the contribution. In #1 "adequately" is a bit vague but I assume you are linking this to cirterion validity in #3. In both cases you need to modify these to recognise that your tests of finger printing and ICC do not constitute the entire literature on test-retest measurement. So perhaps modify #1 as "... where previously proposed statistics fail." -> "... where *several* previously proposed statistics fail." You cannot imply that you have tested all previously proposed statistics. For example, I believe the NPAIRS metrics of Churchill et al., (2015; https://pubmed.ncbi.nlm.nih.gov/26678485/) both "adequately quantifies the relative contribution of various accidental and systematic deviations" and constitute "multivariate measure of reliability that has been explicitly related to criterion validity, e.g., behavioural measures. As a result you cannot claim that Discr is the only measure to have been linked to criterion validity, and in addition, #3 should be modified to acknowledge the limitations on the lower bound on non-Gaussian predictive accuracy. I would also point out that some authors have bypassed the need for an intermediate metric completely to show better preprocessing selections wrt cirterion validity such as age, e.g., Garrett et al., (2010; https://pubmed.ncbi.nlm.nih.gov/20371811/). This brings us back to the importance and generality of the bolded statement on p. 2. How important is it to have an intermediate metric that potentially generalises and avoids at least some post-hoc tests? Is this more than a computational issue given a particular data set? Wrt assessing criterion validity, why should researchers not mainly rely on direct testing as in Garrett and avoid the issue of using intermediate metrics such as Discr, which potentially require their own specially collected test-retest data sets, which may not generalise to subsequent experimental data collections?

P3, #4: "... datasets which preprocessing ..." -> "... datasets with preprocessing ..."

P3, "Second, Discr only considers comparisons of the ranks of pairwise distances between different items with the ranks of pairwise distances between the same item. All other information is literally discarded, as it does not provide insight into the question of interest." This property seems to imply that Discr saturates at 1 and cannot distinguish situations where a particular processing choice might ensure twice as much variance is

captured between items (subjects) provided the within-item distances are small enough for Discr=1. Please explain why, as a matter of experimental design, a researcher would not be interested in choices that significantly increase the between-subject structure that may then be linked to covariate criterion validity when the multiple associated processing choices produce Discr=1?

P4, #4: "... across-subject measurements ..." -> "... across-item measurements ..."

P4. Please provide a full and preferably accessible citation for #34.

P6. "... yield in unacceptably ..." -> "... yield unacceptably ..."

P6. "Thus, prior to embarking on a machine learning search for predictive accuracy, one can simply test whether the data are discriminable at all. If not, predictive accuracy will be hopeless." But discriminability with Discr is associated with between-subject distances and many machine learning searches for predictive accuracy are about differences between subject classes, not between-subject differences. Increasing between-subject effects might reduce between-subject group effects since it may increase within-group, between-subject distances. Please briefly address this issue in a few sentences to back up your claim of generally improved predictive accuracy as opposed to the special case measured by Discr.

P6-7. "Figure 2D shows that Discr achieves approximately the highest power among all competing approaches in all settings and variances", and in the Fig. 2 caption "Discr typically achieves highest power among the alternative statistics in all cases." These seem to be misleading statements when Discr clearly does not achieve the highest power over most of variance range relative to I2C2 and kernel in (iii) and kernel in (iv). Overall Discr seems to be equaled or outperformed by Kernel across all four simulated samples, which is a rather different message, and the statements should be reworded to reflect the reality of the plotted results.

Finally the following statements make me wonder if the figure caption colors are mislabelled. "Non-parametric Discr therefore has the power of parametric approaches for data at which those assumptions are appropriate, and much higher power for other data. Kernel performs comparably to Discr in these settings, though with somewhat less power in row (iv) XOR." The last sentence is exactly the opposite of what I see in the plot where the blue kernel curve is mostly above the red Discr curve. These interpretations and plots need to be resolved.

P8. A similar issue seems to occur for Figure 2E. The statement in the Figure caption for (E) that "Comparison test of which approach is more discriminable. Discr typically achieves highest power for all settings and variances." is simply incorrect as seem in the plots for (i)--where Discr is outperformed by Finger over most of the variance range--and by Kernel in (iv). However, with Figure 2D replaced by Figure 2E on p8, I tend to agree with the more nuanced trade-off description in the para. at the end of Sec. 3.3.

P8, Sec. 3.4: This section provides an impressive example of the Discr metric in an important real-world application about which 100s if not 1000s of papers have been published. I hope the authors plan to explore many of the details of this section in another expanded manuscript as there are many issues they have little time to address wrt the existing literature where their nominally optimal preprocessing choices do not seem to accord with the relevant neuroimaging literature as I understand it. Regardless, I think they have done an excellent job using these relatively comprehensive neuroimaging data sets and test results to make a strong case for the potential utility of Discr.

One important issue that I do not see discussed anywhere is the question of test-retest temporal separation. In highly non-stationary situations such as resting state fMRI the relationship of state to trait effects and their influence on Discr may well depend to a considerable extent on temporal test-retest measurement separation. The authors need to briefly address this somewhere in their manusdcript, e.g., in the Appendix the temporal test-retest structure of the data sets analysed and plotted in Figure 3 should be briefly reported.

P8. "Because each stage is nonlinear, it is possible that the best sequence of choices is not equivalent to the best choices on their own. For this reason, publications that evaluate a given stage using any metric, could result in misleading conclusions if one is searching for the best sequence of steps." This point has been raised multiple times in the neuroimaging literature, which should be noted, e.g., Strother (2006; https://pubmed.ncbi.nlm.nih.gov/16568935/).

P.10. Please make the means of all choices more visible in Fig. 4A and the color key for Fig. 4B seems to be missing.

P. 12. There seems to be no explicit description or Fig. 5(II) either in the text or the Figure 5 caption, e.g., it is not immediately obvious what the colored dots represent.
