# Supplementary material for: Eliminating accidental deviations to minimize generalization error and maximize replicability: Applications in connectomics and genomics
Source: PLoS Comput Biol. 2021 Sep 16;17(9):e1009279. doi: 10.1371/journal.pcbi.1009279 (PMC8500408; doi:10.1371/journal.pcbi.1009279)
Supplement: Attachment — Submitted filename: Discrim Response (1).pdf [file pcbi.1009279.s009.pdf]

The point-by-point is organized as follows. Comments from reviewers are enumerated in black text. Our responses are provided in blue text. Relevant text changes which specifically address the reviewer's comment are indicated by the red text within the screenshot (and the corresponding manuscript).

Submission sent back request

1.) We note that the following authors have not yet confirmed their authorship on this manuscript: Shangsi Wang, Zhi Yang, Zeyi Wang, Ting Xu, Cameron Craddock, Christopher Douville, Carey E. Priebe, Michael Milham, Xi-Nian Zuo.

At this point this request is not urgent; we have re-sent their co-author confirmation email containing an auto-confirmation link for them to click, and we will reach out to them at a later stage if we don't hear from them. We wanted to mention this in case you are aware of them experiencing technical issues with confirming authorship via the link. If so, please do ask them to contact us directly via email and we'd be happy to assist.

We have reached out to close collaborators of one of the authors whom we are not sure of their existing best contact email and are waiting to hear back, but have not reached out to other co-authors at this time as we believe all emails provided were best points of contact for them. Please let us know when you have reached out at a later stage, and we are happy to directly reach out as well to remaining co-authors if responses are not obtained.

2.) Please ensure that the CRediT author contributions listed for every co-author are completed accurately and in full.
At this stage, all of the Authors require contributions. Please ensure that the full contributions of each author are acknowledged in the ""Add/Edit/Remove Authors"" section of our submission form.

The corresponding author contributions have been completed.

3.) We ask that a manuscript source file is provided at Revision. Please upload your manuscript file as a .doc, .docx, .rtf or .tex. If you are providing a .tex file, please upload it under the item type 'LaTeX Source File' and leave your .pdf version as the item type 'Manuscript'.

We have provided the manuscript source file as a .tex file.

4.) Please provide separate figure files in .tif or .eps format only, and remove any figures embedded in your manuscript file. If you are using LaTeX, you do not need to remove embedded figures.
For more information about figure files please see our guidelines:
https://journals.plos.org/ploscompbiol/s/figures

https://journals.plos.org/ploscompbiol/s/figures#loc-file-requirements

We have provided the figure files in .tif format.

5.) Please ensure that all author names are spelled correctly in the manuscript file, including the use of a middle name/initials, and any special characters (accents, umlauts, etc.). Please place first/given names before last/family names. All authors must have a first and last name. We noticed that Joshua T. Vogelstein is spelled differently between the manuscript file and submission form. Please be aware that correct spelling and formatting of author names in the article file is vital for accurate indexing in PubMed.

We have added the “T.” middle initial to Joshua Vogelstein’s Orcid profile.

6.) Please provide an Author Summary. This should appear in your manuscript between the Abstract (if applicable) and the Introduction, and should be 150–200 words long. The aim should be to make your findings accessible to a wide audience that includes both scientists and non-scientists. Sample summaries can be found on our website under Submission Guidelines.

We have provided an author summary, which is 173 words in length.

7.) We notice that your supplementary materials (Appendix A-G) are included in the manuscript file. Please remove them and upload them with the file type 'Supporting Information' . Please ensure that all Supporting Information files are included correctly and that each one has a legend listed in the manuscript after the references list.

We have revised our supporting information to be a file separate from the main content, and have attempted to reflect the standard outlined https://journals.plos.org/ploscompbiol/s/supporting-information. Further, we have added a legend in the back of the main manuscript detailing each of the supporting information subsections.

8.) Please amend your detailed Financial Disclosure statement. This is published with the article, therefore should be completed in full sentences and contain the exact wording you wish to be published.

i) Please list the grants (with grant number) or organizations (with url) that supported your study, including funding received from your institution.
ii). State the initials, alongside each funding source, of each author to receive each grant.
iii). State what role the funders took in the study. If the funders had no role in your study, please state: “The funders had no role in study design, data collection and analysis, decision to publish, or preparation of the manuscript.”
iv). If any authors received a salary from any of your funders, please state which authors and which funders.

We have separated the funding statement into a separate subsection, and have added the disclosure provided in (iii). Further we have clarified that the first list of grants are provided to Joshua T. Vogelstein.

Reviewer #1

I enjoyed reading this manuscript, which introduces a number of important ideas and associated practical analytic techniques to the experimental test-retest literature, and then illustrates their use in an impressive analysis of processing choices in big data settings for neuroimaging and genomics. To achieve this the authors introduce a non-parametric comparison of distance ranks under some metric--Euclidean for the purposes of this manuscript--comparing between- versus within-item pairwise distances as a possible way to address the scientific reproducibility/replication challenge that has been highlighted in many fields of late. The authors calculate this metric Discr as the fraction of all possible pairwise distances in the sample for which the withinitem distances (e.g., within subject) are smaller than between-item distances. As such it is an interesting, nontrivial, nonparametric extension of the classical multi-class (Fisher) discriminant that maximises between versus within variance/covariance structure and it thereby allows for a similar and intuitive interpretation of discriminable items, but in a non-parametric and potentially non-Gaussian setting, i.e., Discr attains the maximum value of 1 when all items are discriminable given relatively small within-item and large between-item distances. They also explore possible nonparametric, non-Gaussian kernel generalisations of the classical multiclass discriminant and show that over their simulations this does not perform as well as Discr. because of the small within samples and large between samples typical of test-retest experiments. I believe this is an important insight that considerably strengthens their scientific contribution.

1. They could spend a little more time discussing the practical pros and cons of their approach as it is not obvious to me that with item=subject in neuroimaging the non-stationarity of human brain signals over time necessarily implies that minimising within-subject pattern distances only up to the point that they are less than between-subject differences is a sufficient metric for most criterion validity. See my comments re. saturation of Discr below, although I recognise that the results in Fig. 5 provide a strong case for the utility of Discr wrt the particular case of criterion validity for subject-dependent covariates.

Thank you for this important point. We have clarified in the manuscript this potential problem (and the below problem, re: saturation of discriminability) to note explicitly that if discriminability is saturated, the discriminability statistic has discarded any other useful information, and therefore would not be of further use for downstream inference in determining which strategy to go with, as the reviewer notes. However, we note that discriminability can be trivially augmented to provide an informative metric, by simply shifting or rescaling the between-item distances to render perfect discriminability arbitrarily difficult to achieve.

Moreover, if multiple strategies are saturated at a perfect discriminability (Discr = 1), it does not provide an informative way to differentiate between these strategies. One could trivially augment the discriminability procedure to compare within-item distances to a scaled and/or shifted transformation of between-item distances, thereby rendering perfect discriminability arbitrarily difficult. With these caveats in mind, Discr remains a key experimental design consideration across a wide variety of settings.

In addition to k-class kernel methods the authors compare the performance of their Discr measure to several existing measures of data reproducibility, namely their multivariate intra-class correlation coefficient (I2C2) and a fingerprinting metric introduced in the functional neuroimaging literature. They do a good job of arguing the benefits of Discr using simulations for which I found the simulations and figures very informative. They also show that in real data, Discr appears to track with "criterion validity" such as ageand sex in functional neuroimaging, although their claim that this is the "the only multivariate measure of reliability that has been explicitly related to criterion validity" is incorrect (see below). Nevertheless, their analysis of fMRI and DTI pipeline choices is impressive and seems relatively comprehensive given the particular preprocessing and analysis choices compared. I am less familiar with the genomic framework analysed but it is a strength of the manuscript that they demonstrate the utility of Discr in two separate problem domains.

2. At places in the manuscript reliability seems to be defined as computation reproducibility and is used interchangeably with general reproducibility. For example on p. 1: " Engineering and operations research have been concerned with reliability for a long time, as they require that their products are reliable under various conditions" and "A different strategy has been to quantify the reproducibility, or reliability, of ones' data ...". It would be helpful to have these terms very clearly defined based on the 2019 report of the National Academies of Sciences, Engineering and Medicine, "Reproducibility and Reliability in Science" (http://nap.edu/25303), and then used in the context of these clear definitions. In addition, the terms repeatability, stability and robustness are also mentioned as being closely related but again no distinguishing definitions are provided.

I think, specifically, we are referring to what the NAS terms "replicability", in most contexts. Specifically, it would appear as though we refer to a special case of a test of replicability, measurement repeatability, which we attempt to clarify as such. The only caveat, we find, is that "test-retest reliability" is a fairly established wording for a specific technique that we believe assesses replicability via measurement repeatability, so we do not attempt to change the literature-accepted name of the technique, especially since the NAS article does not appear to differentiate/define reliability from replicability in an explicitly manner. Where possible, we have attempted to clarify replicability as the high-level concept, of which measurement repeatability is one particular approach we focus on. Please see the listed wording changes in red in the attached document; they are very interspersed, so noting *all* of them in screenshots would likely be prohibitively cumbersome.

3. P2, "... many studies have been devoted to quantifying the reproducibility of different univariate properties of the data [14–17]". This is a highly selective set of mainly recent

references so at a minimum needs to be proceeded by e.g., indicating that a much older and larger literature exists. In particular, there are multiple publications that have considered issues well beyond the basic ICCs that have come to dominate the more recent reliability, test-retest literature, e.g., https://pubmed.ncbi.nlm.nih.gov/10408770/ from 1999. In this sense the improvement of Discr over ICC or even I2C2 is clear but does not constitute all of the neuroimaging literature on test-retest performance.

We appreciate this comment, and have noted that other approaches such as NPAIRS have been proposed for extremely specific neuroimaging contexts, with the limitation that it is unclear how to extend NPAIRS outside of the SPM functional imaging environment, such as to connectome or genomics data.

contexts. A number of other approaches such as NPAIRS [19, 20] provide general frameworks for evaluating activation-based neuroimaging timeseries experiments, but these approaches are limited in their applicability outside of their specific domain and data derivative environment. It is unclear, for instance, how to apply the NPAIRS framework to data which is graph-valued, rather than timeseries-valued. A

4. P2, "Specifically, we advocate for explicitly and specifically designing experiments to ensure that they provide highly reproducible data, rather than hoping that they do and performing post-hoc checks after collecting the data." This sentence seems to imply the techniques to be discussed will require no knowledge of, or post-hoc measurements derived from the experimental data, but Discr can only be calculated given experimental data. Please be clearer about exactly what post-hoc checks you expect to avoid and how these will be avoided? Your bolded sentence seems to promise more than your technique delivers. In fact you note the caveat that no reproducibility metric can guarantee general criterion validity and this rather important limitation should be reflected in other statements throughout the manuscript.

To be more specific, we believe that replicability is a necessary component of experimental design when designing the experimental protocol, and not something to be argued in favor of after the experiment has been designed. Every adjustment must be made in the context as to how the adjustment impacts replicability. No matter what the scientific question, we believe that replicability is critical. We have clarified just after the noted bold statement:

Specifically, **we advocate for explicitly and specifically designing experiments to ensure that they provide highly replicable data, rather than hoping that they do and performing post-hoc checks after collecting the data.** Thus, we concretely recommend that new studies leverage existing protocols that have previously been established to generate highly replicable data. If no such protocols are available for your question, we recommend designing new protocols in such a way that replicability is explicitly considered (and not compromised) in each step of the design. Experimental design has a rich

5. P2. Please provide full references with links for ref. 25 and 26.

We have updated the desired references, which is now [25] -> [27] (since we added a citation to the NAS article):

[illegible]

[25] Liam Paninski. Asymptotic theory of information-theoretic experimental design. Neural Comput., 17(7):1480–1507, Jul 2005. ISSN 0899-7667. doi: 10.1162/0899766053723032.

[26] Lee J. Cronbach, Nageswari Rajaratnam, and Goldine C. Gleser. THEORY OF GENERALIZABILITY: A LIBERALIZATION OF RELIABILITY THEORY. British Journal of Statistical Psychology, 16 (2):137–163, Nov 1963. ISSN 0950-561X. doi: 10.1111/j.2044-8317.1963.tb00206.x.

[27] Stephanie Noble, Marisa N. Spann, Fuyuze Tokoglu, Xilin Shen, R. Todd Constable, and Dustin Scheinost. Influences on the Test-Retest Reliability of Functional Connectivity MRI and its Relationship with Behavioral Utility. Cereb. Cortex, 27(11):5415–5429, Nov 2017. ISSN 1460-2199. doi: 10.1093/cercor/bhx230.

Further, previous [26] was an accidental citation to the wrong Dr. Wang; we have removed the reference and updated it to current [28]:

related to ideas proposed by Cronbach's "Theory of Generalizability" [26, 27]. To do so, we leverage our recently introduced `Discr` statistic [28]. `Discr` quantifies the degree to which multiple measurements

6. P2. The authors claim that finally and most uniquely, " Discr is the only non-parametric multivariate measure of test-retest reliability with formal theoretical guarantees of convergence and upper bounds on subsequent inference performance." I agree with the authors that this is a potentially important feature to have and congratulate the authors on their proof. But this bound is conflated with their statement that "(Discr) it is nonparametric, meaning that its validity and interpretation do not depend on any parametric assumptions, such as Gaussianity." On p. 5 the authors state their "Theorem 1. Under the multivariate Gaussian mixture model plus additive Gaussian noise setting, Discr provides a lower bound on the predictive accuracy of a subsequent classification task. Consequently, a strategy with a higher Discr provably provides a higher bound on predictive accuracy than a strategy with a lower Discr." It seems that their bound only formally exists for multivariate Gaussian models so that they should not claim the existence of the bound independent of "... any parametric assumptions, such as Gaussianity." Please modify these statements to include the appropriate caveats.

The only result that depended on the Gaussian assumption was the mahalanobis criterion in the proof of Theorem 1; i.e., Devijver and Kittler's result (Devijver and Kittler, 1982). A closer reading of the relevant criterion (equation 2.92 in the book) reveals that this bound, in fact, holds for more relaxed conditions, and only in fact requires finite first and second moments for the two classes, which makes this a more general bound than we anticipated. We have revised the multivariate additive gaussian noise setting to the multivariate additive noise setting, and have restructured the proof of Theorem 1 to make this connection more explicit. The remaining requirements such as independence and equal class-conditional covariance remain identical, but hopefully this specification relaxes many of the concerns to make the assumptions at least fairly distribution free (as the class of distributions which have finite mean and covariance is much wider than simply Gaussians). Further, we found a number of other situations in which the bound will also hold, including particular functions of the distances (one-to-one variations of local ordinal embeddings, which preserve the distance rankings and the Bayes error). We have added a new corollary to connect these with discriminability in the appendix.

Assumption 2 (Multivariate Additive Noise Setting).

The multivariate additive noise setting can be described as follows. For items $i = 1, \ldots, n$ and sessions $j = 1, \ldots, s$:

$$\begin{aligned} y_i &\overset{iid}{\sim} Bern(\pi_1), \\ \boldsymbol{\theta}_i &\overset{ind}{\sim} \mathcal{F}(\boldsymbol{\mu}_{y_i}, \boldsymbol{\Sigma}_\theta), \\ \boldsymbol{\epsilon}_i^j &\overset{iid}{\sim} \mathcal{F}(\boldsymbol{c}, \boldsymbol{\Sigma}_\epsilon) \text{ independent of } \boldsymbol{\theta}_i, \\ \boldsymbol{x}_i^j &= \boldsymbol{\theta}_i + \boldsymbol{\epsilon}_i^j = f(g(\boldsymbol{\theta}_i)). \end{aligned}$$

where $\mathcal{F}(\boldsymbol{\mu}, \boldsymbol{\Sigma})$ denotes a distribution with a finite mean vector $\boldsymbol{\mu}$ and a finite, non-singular covariance $\boldsymbol{\Sigma}$.

To obtain a bound on Bayes error, we use the following two observations:

1. The weighted covariance matrix of the measurements is non-singular: Define $\boldsymbol{\Sigma}_x$ as the weighted covariance matrix of $\boldsymbol{x}$:

$$\begin{aligned} \boldsymbol{\Sigma}_x &= \pi_0 \text{Var}(\boldsymbol{x}_i^j | \boldsymbol{y}_i = 0) + \pi_1 \text{Var}(\boldsymbol{x}_i^j | \boldsymbol{y}_i = 1) \\ &= \pi_0 \text{Var}(\boldsymbol{\theta}_i | \boldsymbol{y}_i = 0) + \pi_1 \text{Var}(\boldsymbol{\theta}_i | \boldsymbol{y}_i = 1) + \text{Var}(\boldsymbol{\epsilon}_i^j) \\ &= \boldsymbol{\Sigma}_\theta + \boldsymbol{\Sigma}_\epsilon. \end{aligned}$$

   which follows since $\pi_0 + \pi_1 = 1$. Further, note that since both $\boldsymbol{\Sigma}_\theta$ and $\boldsymbol{\Sigma}_\epsilon$ are finite and non-singular, their sum $\boldsymbol{\Sigma}_x$ is also finite and non-singular.

2. The between-class difference is finite: Denote $\Delta\boldsymbol{\mu}$ to be the difference between the means of the two classes. Since $\boldsymbol{\epsilon}_i^j$ is assumed to be independent of $\boldsymbol{y}_i$:

$$\Delta\boldsymbol{\mu} = \mathbb{E}(\boldsymbol{x}_i^j | \boldsymbol{y}_i = 0) - \mathbb{E}(\boldsymbol{x}_i^j | \boldsymbol{y}_i = 1) = \mathbb{E}(\boldsymbol{\theta}_i | \boldsymbol{y}_i = 0) - \mathbb{E}(\boldsymbol{\theta}_i | \boldsymbol{y}_i = 1).$$

We apply Devijver and Kittler's result [75], from equation (2.93), which gives that:

$$L^* \leq \frac{2\pi_0\pi_1}{1 + \pi_0\pi_1 \Delta\boldsymbol{\mu}^\top \boldsymbol{\Sigma}_x^{-1} \Delta\boldsymbol{\mu}}.$$

7. P3. List of contributions 1-6. In general I am OK with these except where I believe they overstate the contribution. In #1 "adequately" is a bit vague but I assume you are linking this to criterion validity in #3. In both cases you need to modify these to recognise that your tests of finger printing and ICC do not constitute the entire literature on test-retest measurement. So perhaps modify #1 as "... where previously proposed statistics fail." -> "... where several previously proposed statistics fail." You cannot imply that you have tested all previously proposed statistics. For example, I believe the NPAIRS metrics of Churchill et al., (2015; https://pubmed.ncbi.nlm.nih.gov/26678485/) both "adequately quantifies the relative contribution of various accidental and systematic deviations" and constitute "multivariate measure of reliability that has been explicitly related to criterion validity, e.g., behavioural measures.

We have amended the wording to clarify that we have not described the entirety of the test-retest literature, and have modified point #1 accordingly.

1. Demonstrates that `Discr` is a statistic that adequately quantifies the relative contribution of certain accidental and systematic deviations, whereas previously proposed statistics have not.

Further, we have clarified that by “explicitly”, we mean that the statistic has shown to relate to criterion validity under a particular theoretical model (and have changed explicitly -> theoretically). Based on our reading, it appears as though NPairs has been related to criterion validity empirically, but not theoretically.

3. Provides sufficient conditions for `Discr` to provide a lower bound on predictive accuracy. `Discr` is the *only* multivariate measure of reliability that has been theoretically related to criterion validity.

8. As a result you cannot claim that Discr is the only measure to have been linked to criterion validity, and in addition, #3 should be modified to acknowledge the limitations on the lower bound on non-Gaussian predictive accuracy. I would also point out that some authors have bypassed the need for an intermediate metric completely to show better preprocessing selections wrt cirterion validity such as age, e.g., Garrett et al., (2010; https://pubmed.ncbi.nlm.nih.gov/20371811/). This brings us back to the importance and generality of the bolded statement on p. 2. How important is it to have an intermediate metric that potentially generalises and avoids at least some post-hoc tests? Is this more than a computational issue given a particular data set? Wrt assessing criterion validity, why should researchers not mainly rely on direct testing as in Garrett and avoid the issue of using intermediate metrics such as Discr, which potentially require their own specially collected test-retest data sets, which may not generalise to subsequent experimental data collections?

As-per point 6. above, we have removed the direct link to Gaussianity. Further, we agree other metrics may have indicated such a relationship empirically, we do not believe this is a theoretically motivated relationship, and therefore have amended the wording as follows:

3. Provides sufficient conditions for `Discr` to provide a lower bound on predictive accuracy. `Discr` is the *only* multivariate measure of reliability that has been theoretically related to criterion validity, both parametric and non-parametric.

We find that test-retest datasets tend to be prolific in science, and as noted in the discussion limitation paragraph, can be coerced even when researchers do not think they immediately have a test-retest dataset (e.g., in our genomics experiment, our collaborators did not even realize at first that their dataset *was*, in fact, test-retest).

derivative work. For example in brain imaging, a functional Magnetic Resonance Imaging (fMRI) scan consists of tens to thousands of scans of the brain at numerous time points. In this case, the image can be broken into identical-width time windows to coerce a dataset in which discriminability can be investigated. In another example taken directly from the cancer genomics experiment below, a genomics

9. P3, #4: "... datasets which preprocessing ..." -> "... datasets with preprocessing ..."

What we intended here was illustrates [...] which (of the) preprocessing pipelines maximize Discr. To rectify this lack of clarity, we have amended the sentence structure slightly, to be clear about what is being illustrated more explicitly.

4. Illustrates on 28 neuroimaging datasets from Consortium for Reliability and Reproducibility (CoRR) [30] and two genomics datasets (i) which preprocessing pipelines which maximize `Discr`, and (ii) that maximizing `Discr` is significantly associated with maximizing the amount of information about multiple covariates, in contrast to other related statistics.

10. P3, "Second, Discr only considers comparisons of the ranks of pairwise distances between different items with the ranks of pairwise distances between the same item. All other information is literally discarded, as it does not provide insight into the question of interest." This property seems to imply that Discr saturates at 1 and cannot distinguish situations where a particular processing choice might ensure twice as much variance is captured between items (subjects) provided the within-item distances are small enough for Discr=1. Please explain why, as a matter of experimental design, a researcher would not be interested in choices that significantly increase the between-subject structure that may then be linked to covariate criterion validity when the multiple associated processing choices produce Discr=1?

We thank the reviewer for this point. We hope this point was clarified in our response to point 4.

11. P4, #4: "... across-subject measurements ..." -> "... across-item measurements ..."

We appreciate this comment to keep the wording consistent.

4. *Discriminability is defined as fraction of times across-item measurements are smaller than within-item measurements:* $Discr = 1 - \frac{f}{N(N-1)-g}$.

12. P4. Please provide a full and preferably accessible citation for #34.

Thank you for this point; this citation has been changed to [37] with the new citations added, and has been updated with the accessible DOI:

[37] Zeyi Wang, Haris I. Sair, Ciprian Crainiceanu, Martin Lindquist, Bennett A. Landman, Susan Resnick, Joshua T. Vogelstein, and Brian Caffo. On statistical tests of functional connectome fingerprinting. Can. J. Stat., 49(1):63–88, Mar 2021. ISSN 0319-5724. doi: 10.1002/cjs.11591.

13. P6. "... yield in unacceptably ..." -> "... yield unacceptably ..."

Thank you for this point; we have made the change below.

different from one another in predictable ways, that is, are discriminable. If not, the same assay applied to the same individual on multiple trials could yield unacceptably highly variable results. Thus, prior to

14. P6. "Thus, prior to embarking on a machine learning search for predictive accuracy, one can simply test whether the data are discriminable at all. If not, predictive accuracy will be hopeless." But discriminability with Discr is associated with between-subject distances and many machine learning searches for predictive accuracy are about differences between subject classes, not between-subject differences. Increasing between subject effects might reduce between-subject group effects since it may

increase within-group, between subject distances. Please briefly address this issue in a few sentences to back up your claim of generally improved predictive accuracy as opposed to the special case measured by Discr.

This is an excellent point, and one which we are actively seeking to understand. To this end, we have identified that discriminability can be decomposed into within and between-class discriminabilities using rather trivial applications of the definition of conditional probability and the law of total probability, so that this problem can be examined in isolation. We have added a brief description of our current understanding of this framing of the problem in an appendix section G, and have highlighted this as a future direction of the discriminability framework in the main body.

dependent on age (such as the connectome). Additionally, discriminability can be decomposed into within and between-class discriminabilities, so that class-specific effects may be examined in isolation, as described in Appendix G. Future work could explore how these two quantities may be incorporated into the experimental design procedure.

15. P6-7. "Figure 2D shows that Discr achieves approximately the highest power among all competing approaches in all settings and variances", and in the Fig. 2 caption "Discr typically achieves highest power among the alternative statistics in all cases." These seem to be misleading statements when Discr clearly does not achieve the highest power over most of variance range relative to I2C2 and kernel in (iii) and kernel in (iv). Overall Discr seems to be equaled or outperformed by Kernel across all four simulated samples, which is a rather different message, and the statements should be reworded to reflect the reality of the plotted results. Finally the following statements make me wonder if the figure caption colors are mislabelled. "Nonparametric Discr therefore has the power of parametric approaches for data at which those assumptions are appropriate, and much higher power for other data. Kernel performs comparably to Discr in these settings, though with somewhat less power in row (iv) XOR." The last sentence is exactly the opposite of what I see in the plot where the blue kernel curve is mostly above the red Discr curve. These interpretations and plots need to be resolved. P8. A similar issue seems to occur for Figure 2E. The statement in the Figure caption for (E) that "Comparison test of which approach is more discriminable. Discr typically achieves highest power for all settings and variances." is simply incorrect as seem in the plots for (i)--where Discr is outperformed by Finger over most of the variance range--and by Kernel in (iv). However, with Figure 2D replaced by Figure 2E on p8, I tend to agree with the more nuanced trade-off description in the para. at the end of Sec. 3.3.

We appreciate this note. The purpose of this figure was not to highlight that discriminability was the best for individual columns/rows, but rather, the only statistic with high power in both one and two class, across all simulation settings. As you have noted, we have found it difficult to accurately capture this nuanced tradeoff, since we are effectively attempting to summarize 8 plots across 4 settings. We have weakened our claim that discriminability has the “highest” power and replaced the wording with “high” (it is in the better half for everything, and it is never the case that any statistic achieves high power but Discr does not), and have removed the note about the XOR setting with respect to Kernel in the one-sample regime. Further, we clarify that

discriminability was the only statistic able to achieve high power for the two-sample setting, in which any of the statistics were able to achieve reasonably high power. For instance, Kernel totally fails for the Gaussian case (row (i)), and as noted in the manuscript, Finger as higher power in Gaussian but no power for cross.

Figure 2D shows that Discr achieves high power among all competing approaches in all settings and variances. This result demonstrates that despite the fact that Discr does not rely on Gaussian assumptions, it still performs nearly as well or better than parametric methods when the data satisfy these assumptions (row (i)). In row (ii) cross, only Discr and Kernel correctly identify that items differ from one another, despite the fact that the data are Gaussian, though they are not spherically symmetric gaussians. In both rows (iii) ball/disc and (iv) XOR, most statistics perform well despite the non-Gaussianity of the data. And when there is no signal, all tests are valid, achieving power less than or equal to the critical value. Non-parametric Discr therefore has the power of parametric approaches for data at which those assumptions are appropriate, and much higher power for other data. Kernel performs comparably to Discr in these settings.

Figure 2: **Multivariate simulations demonstrate the value of optimizing reliability for experimental design**. All simulations are two-dimensional, with 128 samples, with 500 iterations per setting (see Appendix D for details). **(A)** For each setting, class label is indicated by shape, and color indicates item identity. **(B)** Euclidean distance matrix between samples within each simulation setting. Samples are organized by item. Simulation settings in which items are discriminable tend to have a block structure where samples from the same item are relatively similar to one another. **(C)** Reproducibility statistic versus variance. Here, we can compute the Bayes accuracy (the best one could perform to predict class label) as a function of variance. Discr and Kernel are mostly monotonic relative to within-item variance across all settings, suggesting that one can predict improved performance via improved Discr. **(D)** Test of whether data are discriminable. Discr typically achieves high power among the alternative statistics in all cases. **(E)** Comparison test of which approach is more discriminable. Discr is the only statistic which achieves high power in all settings in which any statistic was able to achieve high power.

A test to compare reliabilities Given two experimental designs—which can differ either by acquisition and/or analysis details—are the measurements produced by one method more discriminable than the other? Figure 2D shows Discr typically achieves the highest power among all statistics considered. Specifically, only Fingerprint achieves higher power in the Gaussian setting, but it achieves almost no power in the cross setting. Kernel achieves comparably lower power for most settings and no power for the Gaussian, as does PICC. I2C2 achieves similar power to Discr only for the Gaussian and ball/disc setting. All tests are valid in that they achieve a power approximately equal to or below the critical value when there is no signal. Note that these comparisons are not the typical "k-sample comparisons" with many theoretical results, rather, they are comparing across multiple disparate k-sample settings. Thus, in general, there is a lack of theoretical guarantees for this setting. Nonetheless, the fact that Discr achieves nearly equal or higher power than the statistics that build upon Gaussian methods, even under Gaussian assumptions, suggests that Discr will be a superior metric for optimal experimental design in real data.

16. P8, Sec. 3.4: This section provides an impressive example of the Discr metric in an important real-world application about which 100s if not 1000s of papers have been published. I hope the authors plan to explore many of the details of this section in another expanded manuscript as there are many issues they have little time to address wrt the existing literature where their nominally optimal preprocessing choices do not seem to accord with the relevant neuroimaging literature as I understand it. Regardless, I

think they have done an excellent job using these relatively comprehensive neuroimaging data sets and test results to make a strong case for the potential utility of Discr. One important issue that I do not see discussed anywhere is the question of test-retest temporal separation. In highly non-stationary situations such as resting state fMRI the relationship of state to trait effects and their influence on Discr may well depend to a considerable extent on temporal test-retest measurement separation. The authors need to briefly address this somewhere in their manuscript, e.g., in the Appendix the temporal test-retest structure of the data sets analysed and plotted in Figure 3 should be briefly reported.

We have added the temporal test-retest structure to analyzed datasets, where such information was made available, in the Appendix tables:

Dataset	Manuf.	Model	TE (ms)	TR (ms)	STC	#Timepts	#Sub	#Ses	#Scans	TRT (days)	Discr
KKI2009	Philips	Achieva	30	2000	seq.	210	21	2	42	<1	0.93
NKI24	Siemens	TrioTim	30	645	inter.	900	24	2	47	<14	0.98
BNU1	Siemens	TrioTim	30	2000	inter.	200	50	2	100	42	0.97
BNU2	Siemens	TrioTim	30	variable	inter.	variable	50	2	100	103	0.92
DC1	Philips	NA	35	2500	inter.	120	114	4	244	?	0.95
HNU1	GE	MR750	30	2000	inter.	300	30	10	300	3	0.98
IACAS	GE	Signa	30	2000	inter.	240	28	3	59	42	0.83
IBATRT	Siemens	TrioTim	30	1750	seq.	220	36	2	50	None	0.95
IPCAS	NA	NA	NA	NA	NA	NA	78	2	156	–	0.99
IPCAS1	Siemens	TrioTim	30	2000	inter.	205	30	2	60	7	1.00
IPCAS2	Siemens	TrioTim	30	2500	inter.	212	35	2	70	30	0.98
IPCAS5	Siemens	TrioTim	30	2000	inter.	170	22	2	44	>10 (min)	0.96
IPCAS6	Siemens	TrioTim	30	2500	inter.	242	2	15	30	3 (hrs)	1.00
IPCAS8	Siemens	TrioTim	30	2000	inter.	240	13	2	26	>1 (years)	0.96
JHNU	Siemens	TrioTim	30	2000	inter.	250	30	2	60	NA	0.96
LMU3	Siemens	TrioTim	30	3000	inter.	120	25	2	50	NA	0.93
MRN1	NA	NA	NA	NA	NA	NA	53	2	88	120	0.94
NYU1	Siemens	Allegra	25	2000	NaN	197	25	3	75	5-11 (mo)*	0.98
NYU2	Siemens	Allegra	15	2000	inter.	180	187	3	252	<1 (hrs)	0.96
SWU1	Siemens	TrioTim	30	2000	inter.	240	20	3	59	NA	0.97
SWU2	Siemens	TrioTim	30	2000	inter.	300	27	2	54	NA	0.96
SWU3	Siemens	TrioTim	30	2000	inter.	242	24	2	48	NA	0.98
SWU4	Siemens	TrioTim	30	2000	inter.	242	235	2	467	1 (yrs)	0.97
UM	Siemens	TrioTim	30	2000	seq.	150	80	2	160	NA	0.99
UPSM1	Siemens	TrioTim	29	1500	seq.	200	100	3	230	473 - 1434	0.89
Utah1	Siemens	TrioTim	28	2000	inter.	240	26	2	52	>2 (yrs)	0.92
UWM	GE	MR750	25	2600	inter.	231	25	2	50	NA	0.96
XHCUMS	Siemens	TrioTim	30	3000	inter.	124	24	5	120	180	0.91

Figure 6: **fMRI Dataset Descriptions**. In the above table, STC corresponds to slice timing correction. Rows with NA entries do not have available metadata associated with the scanning protocol. The column TRT indicates the follow up time for retest. A value of None indicates that the scans were back to back. The sample Discr corresponds to the Discr of the best performing pipeline overall, FNNNCP. *The test-retest structure for NYU1 was 5 - 11 months between sessions 1 and 2, and $30 - 45$ minutes between sessions 2 and 3.

Dataset	Manuf.	Model	TE (ms)	TR (ms)	#Dir	bval $\frac{s}{mm^2}$	#Sub	#Ses	#Scans	TRT (days)	Discr
BNU1	Siemens	TrioTim	89	8000	30	1000	57	2	113	42	1.00
HNU1	GE	MR750	Min	8600	33	1000	30	10	300	3	0.99
KKI2009	Philips	NA	32	6281	65	700	21	2	42	<1	1.00
NKI24	Siemens	TrioTim	95	2400	137	1500	20	2	40	<14	1.00
SWU4	Siemens	TrioTim	NaN	NaN	93	1000	227	2	454	1 (yrs)	0.88

Figure 7: **dMRI Dataset Descriptions**. In the above table, #Dir corresponds to the number of diffusion directions. Rows with NA entries do not have available metadata associated with the scanning protocol. The sample Discr correspond to the Discr of the pipeline with the CPAC200 parcellation and the log-transformed edges.

Further, we have made explicit note of these tables in the Appendix, by pointing readers to their location in the main body of the text:

Human brain imaging data acquisition and analysis Consortium for Reliability and Reproducibility (CoRR) [39] has generated functional, anatomical, and diffusion magnetic resonance imaging (dMRI) scans from $>$1,600 participants, often with multiple measurements, collected through 28 different datasets (22 of which have both age and sex annotation) spanning over 20 sites. Each of the sites use different scanners, technicians, scanning protocols, and retest follow up procedures, thereby representing a wide variety of different acquisition settings with which one can test different analysis pipelines. Appendix F.3 provides protocol metadata associated with each individual dataset. Figure 3A [illegible]

17. P8. "Because each stage is nonlinear, it is possible that the best sequence of choices is not equivalent to the best choices on their own. For this reason, publications that evaluate a given stage using any metric, could result in misleading conclusions if one is searching for the best sequence of steps." This point has been raised multiple times in the neuroimaging literature, which should be noted, e.g., Strother (2006; https://pubmed.ncbi.nlm.nih.gov/16568935/).

We agree this point has been raised, and have cited the indicated article.

analysis pipelines. Because each stage is nonlinear, it is possible that the best sequence of choices is not equivalent to the best choices on their own. For this reason, publications that evaluate a given stage using any metric, could result in misleading conclusions if one is searching for the best sequence of steps [43]. The dMRI connectomes were acquired via 48 analysis pipelines using the Neurodata MRI

18. P.10. Please make the means of all choices more visible in Fig. 4A and the color key for Fig. 4B seems to be missing.

We have updated the plot and the caption to reflect that the mean is now shown in red, and that the color caption for 4B corresponds to the color caption for Fig 3, as in Fig 5.

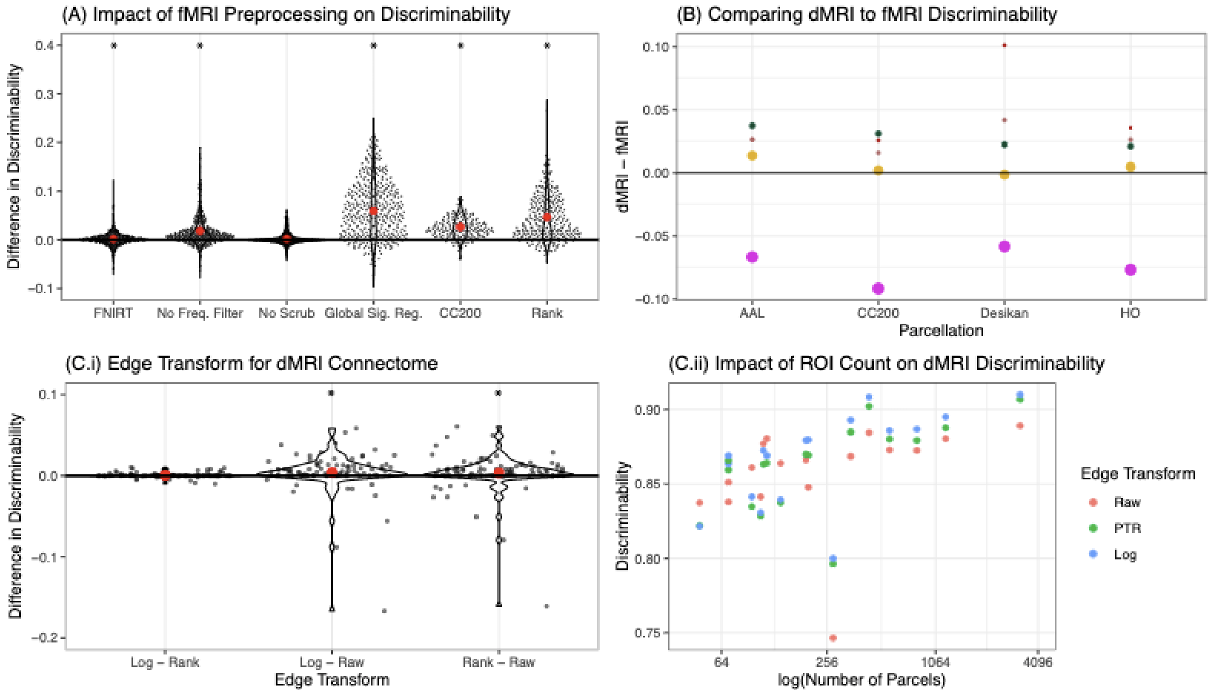

Figure 4: **Parsing the relative impact on Discr of various acquisition and analytic choices**. **(A)** The pipelines are aggregated for a particular analysis step, with pairwise comparisons with the remaining analysis options held fixed. The beeswarm plot shows the difference between the overall best performing option and the second best option for each stage (mean in red) with other options held equal; the x-axis label indicates the best performing strategy. The best strategies are FNIRT, no frequency filtering, no scrubbing, global signal regression, the CC200 parcellation, and ranks edge transformation. A Wilcoxon signed-rank test is used to determine whether the mean for the best strategy exceeds the second best strategy: a * indicates that the p-value is at most 0.001 after Bonferroni correction. Of the best options, only no scrubbing is *not* significantly better than alternative strategies. Note that the options that perform marginally the best are not significantly different than the best performing strategy overall, as shown in Figure 3. **(B)** A comparison of the stabilities for the 4 datasets with both fMRI and dMRI connectomes. dMRI connectomes tend to be more discriminable, in 14 of 20 total comparisons. Color and point size correspond to the study and number of scans, respectively (see Figure 3B). **(C.i)** Comparing raw edge weights (Raw), ranking (Rank), and log-transforming the edge-weights (Log) for the diffusion connectomes, the Log and Rank transformed edge-weights tend to show higher Discr than Raw. **(C.ii)** As the number of ROIs increases, the Discr tends to increase.

To this end, we have also made the means more obvious in Figure 3B as well, by adding a slight alpha to the colored points, and increasing the triangle size (while decreasing point size):

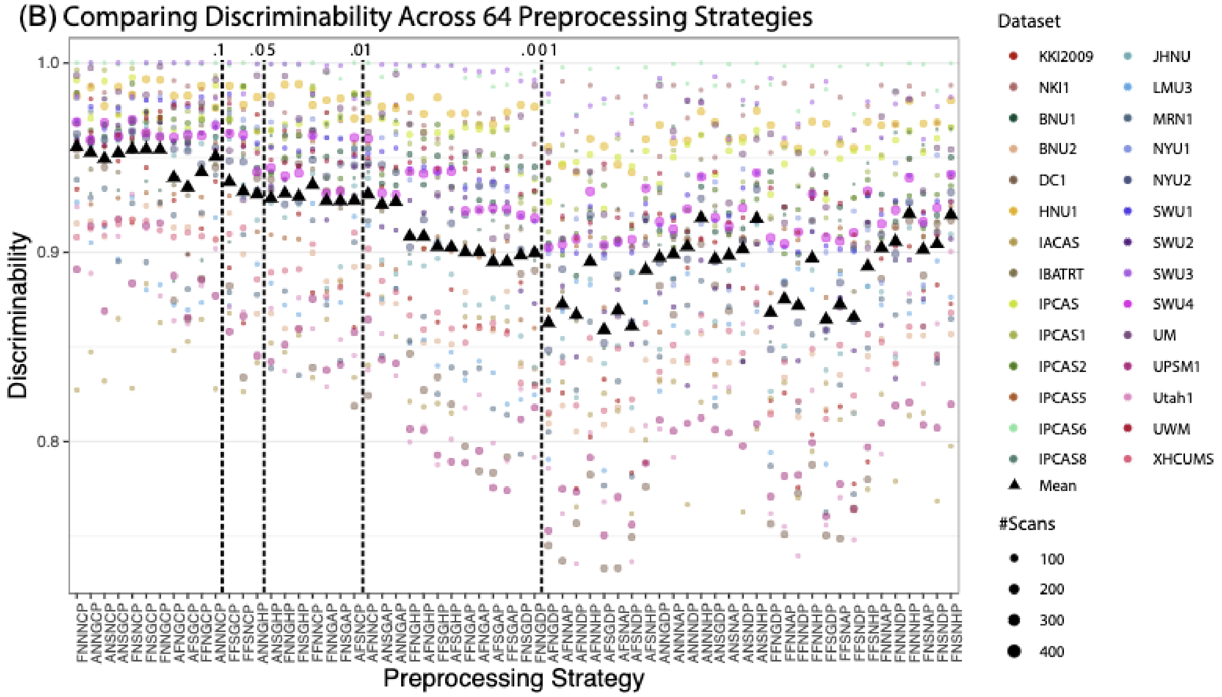

19. P. 12. There seems to be no explicit description or Fig. 5(II) either in the text or the Figure 5 caption, e.g., it is not immediately obvious what the colored dots represent.

Thank you for this clarification. We have added a legend for Fig. 5(II).

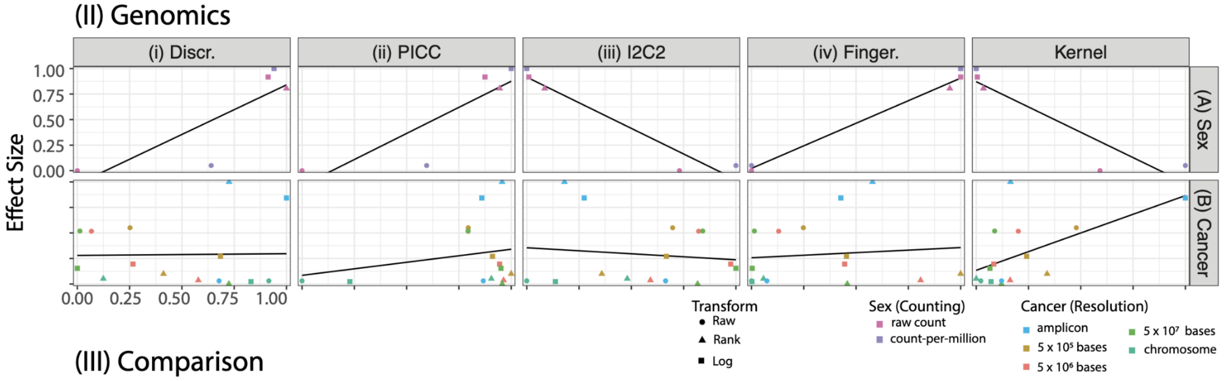

(III) Comparison

Reviewer #3

Spurred by the reproducibility issue, and motivated by classical works in statistics (Spearman, Fisher), the authors expand on a statistic called discriminability, which quantifies the degree to which an individual's samples are similar to one another. (Some of the authors proposed this index earlier.) The aim is to minimize generalization error and maximize reliability. Discriminability is a nonparametric index that is appropriate for a wide range of data types (text, images). By using simulations, proving theorems (for additive noise), and demonstrating its use on neuroimaging and genomic data, they show that their index outperforms intraclass correlation (ICC), fingerprinting index, and kernel methods.

Comments:

1. The authors say that the ICC's Gaussian roots are a problem. However, their theorems deal with Gaussian errors (the mixture of Gaussians seems fine, for they are flexible). What happens if the errors are not Gaussian ? Many homogeneous populations are skewed, so skew-normal or related distributions may be more appropriate.

We would like to thank the reviewer for these points. We believe we have addressed this limitation in our response to reviewer #1, point 6, and have generalized to distributions with finite mean and covariance, or sufficient generalizations thereof. Skew-normal distributions would be a particular special case of our result.

Minor Comments:

The paper is very well written; I spotted a few awkward phrasings

2. why SUM i * s_{i} ? why not SUM s_{i} ?

Thank you for this catch. Good point.

Definition 1 (Inter-Item Discriminability). *Assuming we have n items, where each item has s_i measurements, we obtain $N = \sum_{i=1}^{n} s_i$ total measurements. For simplicity, assume $s_i = 2$ for the definition below, and that there are no ties. Given that, $\texttt{Discr}$ can be computed as follows (for a more formal definition and pseudocode, please see Appendix B):*

3. indentify —> identify

I cannot find this wording in the document at present; we must have remedied it on a read over in the last month or two.

4. for the below definition —> for the definition below

Thank you for catching this; we have shown this correction in point 2.

5. to overcome to requirement of ICC —> to overcome the requirement of ICC

We appreciate this suggestion, and have made the fix as shown below.

Numerous multivariate generalizations of the ICC attempt to overcome the requirement of ICC to operate on univariate data. The Image Intra-Class Correlation (I2C2) was introduced to mitigate ICC's
